# Bioinspired Nucleophilic Attack on a Tungsten-Bound Acetylene: Formation of Cationic Carbyne and Alkenyl Complexes

**DOI:** 10.1021/acs.inorgchem.1c00643

**Published:** 2021-04-14

**Authors:** Madeleine A. Ehweiner, Lydia M. Peschel, Niklas Stix, Miljan Z. Ćorović, Ferdinand Belaj, Nadia C. Mösch-Zanetti

**Affiliations:** Institute of Chemistry, Inorganic Chemistry, University of Graz, 8010 Graz, Austria

## Abstract

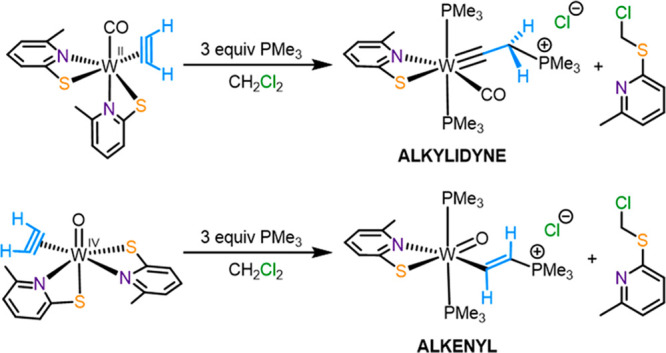

Inspired by the proposed inner-sphere mechanism of the tungstoenzyme acetylene hydratase, we have designed tungsten acetylene complexes and investigated their reactivity. Here, we report the first intermolecular nucleophilic attack on a tungsten-bound acetylene (C_2_H_2_) in bioinspired complexes employing 6-methylpyridine-2-thiolate ligands. By using PMe_3_ as a nucleophile, we isolated cationic carbyne and alkenyl complexes.

## Abstract 2

We report the first intermolecular nucleophilic attack on a tungsten-bound C_2_H_2_ in two bioinspired complexes differing only by the oxidation state of the metal center and one ligand. By using PMe_3_ as a nucleophile, we isolated cationic carbyne and alkenyl complexes.

The anaerobic bacterium *Pelobacter acetylenicus* can grow on acetylene (C_2_H_2_) as a single carbon and energy source. Its utilization is performed by the tungstoenzyme acetylene hydratase (AH), which catalyzes the hydration of acetylene to acetaldehyde.^1−4^ The coordination sphere of the tungsten(IV) center in the active site consists of four sulfur atoms from two molybdopterin cofactors, a thiolate from cysteine, and a water molecule.^5^ Although several experimental^5,6^ and computational studies^7−10^ have been carried out to shed light on the reaction mechanism, it remains unclear where C_2_H_2_ is located during hydration. Because of lower energetic barriers, density functional theory calculations favor a mechanism where C_2_H_2_ replaces the coordinated water and subsequently undergoes nucleophilic attack by a hydroxide (Scheme 1).^7−10^ Apart from investigations on [Et_4_N]_2_[WO(mnt)_2_] (mnt = maleonitriledithiolate),^11−13^ synthetic approaches to elucidate the mechanism of AH have only included systems with tungsten-coordinated C_2_H_2_.^14,15^ In a recent publication, the S,N-bidentate ligand pyridine-2-thiolate (PyS) was introduced to the tungsten(II) center to model the active site of AH as in Inter1 (Scheme 1).^15^ In contrast to previously reported structural model compounds,^14^ the coordination of a second C_2_H_2_ and subsequent insertion into the W–N bond occurred, showing that a second coordinated C_2_H_2_ is sufficiently activated to undergo a nucleophilic attack. A similar behavior was observed in molybdenum and tungsten complexes, where a nucleophilic attack on one of two coordinated hexafluorobut-2-yne moieties took place, yielding a η^2^-vinyl complex.^16−20^ Our aim is to facilitate an intermolecular nucleophilic attack by inhibiting the coordination of a second C_2_H_2_ and insertion as in the PyS system. Therefore, we anticipated the introduction of a methyl group next to the coordinating nitrogen atom in PyS (6-MePyS) so that the metal center is more shielded against the coordination of a second C_2_H_2_.

**Scheme 1:**
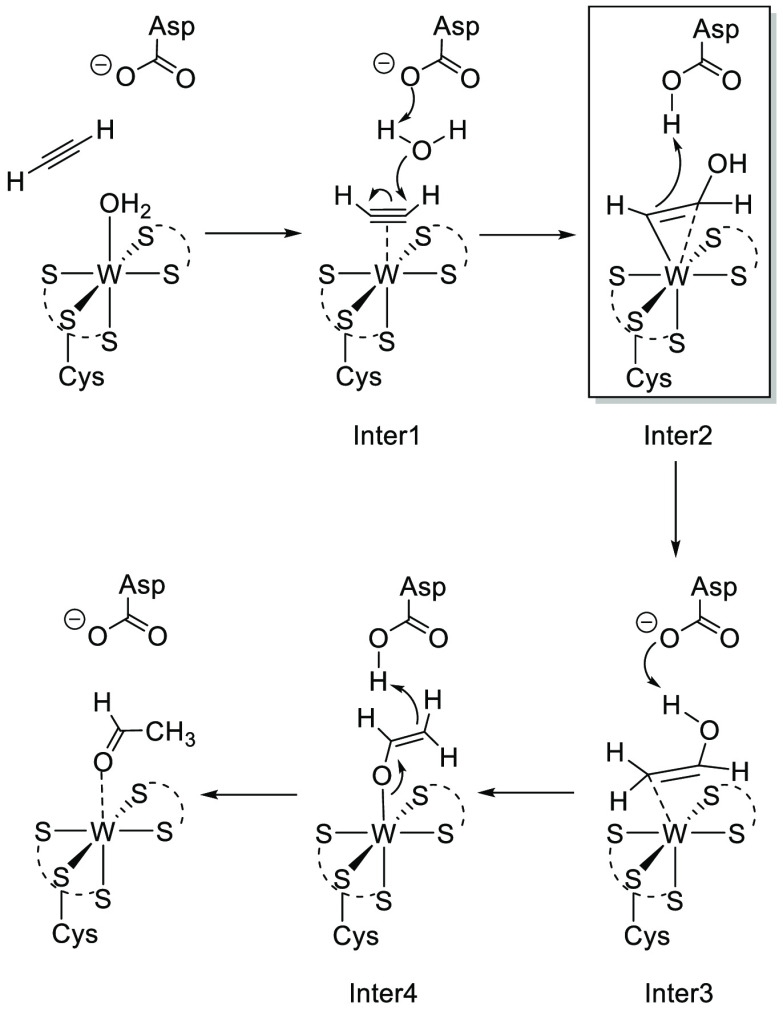
Proposed Inner-Sphere Mechanism of the Hydration of C_2_H_2_ Performed by AH: Nucleophilic Attack of a Hydroxide on Coordinated C_2_H_2_^8^

For preparation of the redesigned tungsten complex [W(CO)(C_2_H_2_)(6-MePyS)_2_] (**1**), which contains the desired 6-MePyS ligands and only one C_2_H_2_, a previously developed procedure was modified.^14,15,21^ The reaction of [WBr_2_(CO)_3_(NCMe)_2_] with 2.1 equiv of Na(6-MePyS) in CH_2_Cl_2_ followed by stirring under a C_2_H_2_ atmosphere for 1 h allowed the isolation of **1** in 86% yield after silica gel filtration. The intermediately formed tricarbonyl complex [W(CO)_3_(6-MePyS)_2_] was characterized by single-crystal X-ray diffraction analysis (see the Supporting Information). Longer reaction times led to the insertion of a second C_2_H_2_ into the W–N bond forming [W(CO)(C_2_H_2_)(HCCH-6-MePyS)(6-MePyS)] (**2**), as previously observed in the unsubstituted analogue [W(CO)(C_2_H_2_)(HCCH-PyS)(PyS)].^15^ However, the additional methyl group significantly decreases intramolecular insertion because even after 24 h and repeated addition of C_2_H_2_ only partial conversion is observed. Furthermore, in the absence of additional C_2_H_2_, **2** reacts reversibly to **1** under the elimination of acetylene or polyacetylene depending on the solvent. Nevertheless, we were able to isolate **2** and unambiguously confirm its structure by single-crystal X-ray diffraction analysis (Figure 1) and by spectroscopic means. In CD_2_Cl_2_, the sterically hindered C_2_H_2_ protons of **1** resonate at 13.77 and 12.50 ppm and the carbon atoms at 205.73 and 204.14 ppm, suggesting that C_2_H_2_ acts as a four-electron donor.^22^ In ^1^H NMR spectra of **2** recorded in CD_2_Cl_2_, the η^2^-C_2_H_2_ protons appear as singlets at 12.90 and 12.03 ppm. The protons of the inserted C_2_H_2_ couple with each other thus appear as doublets (^3^*J* = 10.9 Hz) flanked with ^183^W satellites at 7.61 and 6.89 ppm. IR spectra of **1** and **2** show strong C≡O bands at 1891 and 1897 cm^–1^, respectively. Single-crystal X-ray diffraction analyses of **1** and **2** revealed almost identical W–C1, W–C2, and C1–C2 bond distances (Figure 1) compared to the literature values of tungsten(II) acetylene complexes.^23,24^ The inserted C_2_H_2_ in **2** is strongly activated and therefore has more ethylene character with a C–C bond length of 1.349(3) Å compared to 1.310(3) Å in η^2^-acetylene. Compared to the unsubstituted analogue [W(CO)(C_2_H_2_)(PyS)_2_] with W–N distances of 2.161 and 2.212 Å, **1** exhibits slightly longer bonds [2.197(3) and 2.259(4) Å].^15^ As was already observed in [MoO_2_(6-MePyS)_2_], the nitrogen atom in 6-MePyS is not able to bind to the metal center as tightly as it does in the unsubstituted version because of the methyl group in the ortho position.^25^

**Figure 1:**
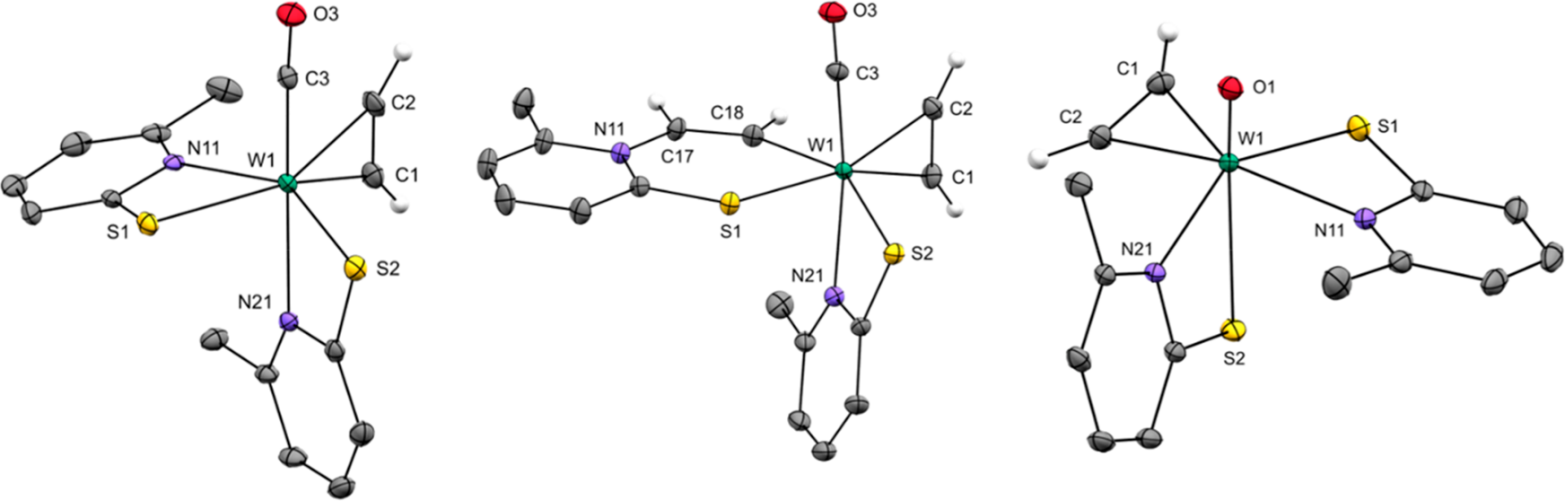
Molecular structures of **1** (left), **2** (middle), and **3** (right) with probability ellipsoids drawn at the 50% probability level.

Considering that the tungsten center in AH is in the oxidation state +IV, we oxidized **1** with pyridine *N*-oxide to obtain the tungsten(IV) complex [WO(C_2_H_2_)(6-MePyS)_2_] (**3**) according to Scheme 2. After filtration to remove insoluble byproducts, **3** was crystallized in 84% yield. IR spectra show one strong band indicative of ν(W=O) at 924 cm^–1^.^14,26−28^ The ^1^H NMR spectrum of **3** in CD_2_Cl_2_ shows two equally sharp singlets flanked with ^183^W satellites for the C_2_H_2_ protons at 11.23 and 10.99 ppm. Thus, they are shifted upfield compared to **1**. The same trend is observed in the ^13^C NMR spectrum, with resonances at 159.69 and 159.05 ppm being characteristic of a two- or three-electron-donor alkyne.^29^ Single crystals suitable for X-ray diffraction analysis were grown from a CH_2_Cl_2_/heptane solution. A molecular view of **3** is displayed in Figure 1. The C1–C2 bond [1.279(2) Å] is slightly shorter than that in **1** [1.306(7) Å], while the W–C bonds [W1–C1 of 2.022(5) Å for **1** vs 2.0693(15) Å for **3** and W1–C2 of 2.055(3) Å for **1** vs 2.1027(15) Å for **3**] are essentially longer.

**Scheme 2:**
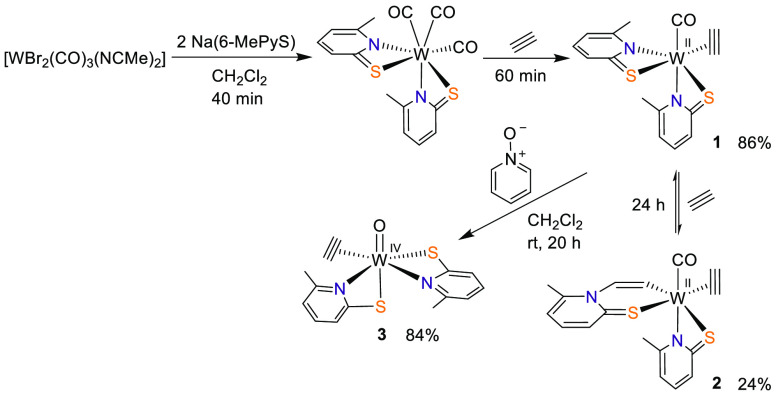
Synthesis of Complexes **1**–**3**

With compounds **1** and **3** exhibiting related structures but different oxidation states of the metal center, a nucleophilic attack of PMe_3_ on the coordinated C_2_H_2_ was investigated. Of particular interest to us was the potential formation of the intermediate Inter2 (Figure 1). Treatment of a CH_2_Cl_2_ solution of **1** with 3 equiv of PMe_3_ led to an immediate color change from purple to orange-brown and full conversion of the starting material. An X-ray diffraction study on single crystals grown from a CH_2_Cl_2_/heptane solution revealed the product to be an ion pair consisting of the tungsten carbyne complex [W(CO)(CCH_2_PMe_3_)(PMe_3_)_2_(6-MePyS)]^+^ (**4**) and a chloride deriving from the solvent (Figure 2). Indeed, a nucleophilic attack of PMe_3_ at the coordinated C_2_H_2_ under the formation of a P–C bond had occurred. However, the attack also leads to the cleavage of one 6-MePyS, which reacts with CH_2_Cl_2_ to form 2-((chloromethyl)thio)-6-methylpyridine (6-MePySCH_2_Cl) and Cl^–^. The coordination surrounding of tungsten is completed by two PMe_3_ ligands preserving the 18e^–^ character of the complex and explaining the need for 3 equiv of phosphine. After workup, pure **4** was obtained in 50% yield (Scheme 3).

**Scheme 3:**
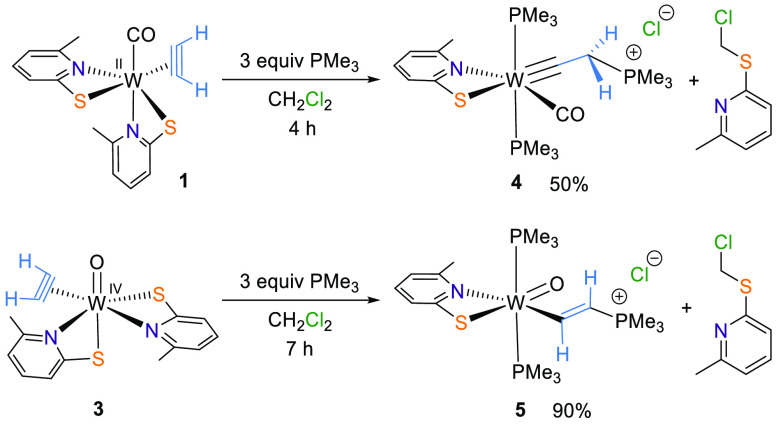
Reaction of **1** and **3** with 3 equiv of PMe_3_ in CH_2_Cl_2_ to Yield **4** and **5**, Respectively

**Figure 2:**
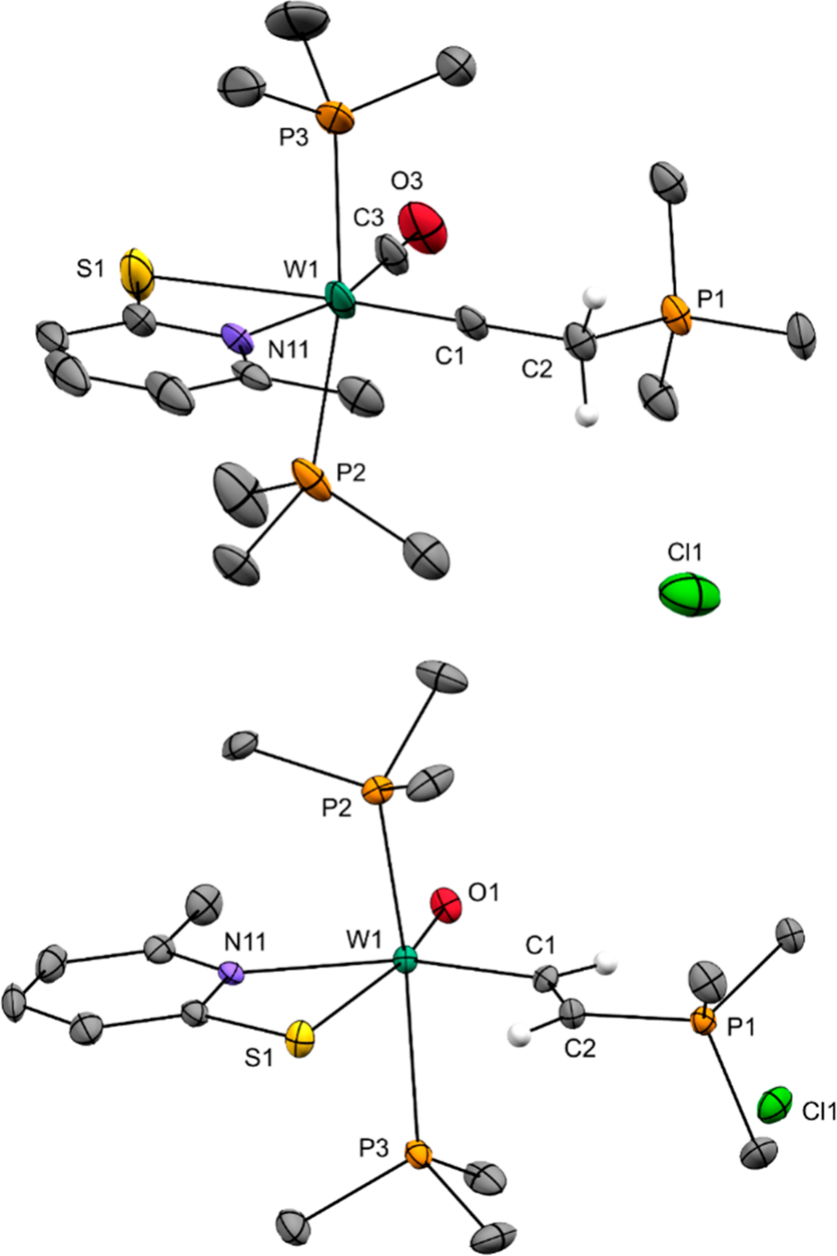
Molecular structures of **4** (top) and **5** (bottom) with probability ellipsoids drawn at the 30% (**4**) and 50% (**5**) probability levels, respectively.

The formation of **4** is also confirmed by ^1^H NMR spectroscopy, where the CH_2_ protons appear at 3.81 ppm as a doublet of triplets (^2^*J*_HP_ = 19.4 Hz; ^4^*J*_HP_ = 5.9 Hz) and the methyl groups of the two tungsten-coordinated PMe_3_ molecules at 1.44 ppm and those of the carbon-bound PMe_3_ at 2.12 ppm. To confirm that the carbyne carbon atom as well as the adjacent methylene group indeed derive from the coordinated C_2_H_2_, **1** was resynthesized using C_2_D_2_ to generate [W(CO)(C_2_D_2_)(6-MePyS)_2_] (**1D**). Upon reaction of **1D** with PMe_3_, the signal at 3.81 ppm is absent, confirming a reaction of the coordinated C_2_H_2_ and no participation of CH_2_Cl_2_.^30^ While Cummins et al. reported the reaction of a molybdenum-bound (trimethylsilyl)acetylene with Li[BHEt_3_] to a η^2^-vinyl complex and the subsequent formation of carbyne only after heating to 80 °C for several hours, the η^2^-vinyl intermediate does not seem to be stable at all in our case.^31^ It is only observed in the ^1^H NMR spectrum when a reaction of **1** is carried out with less than 3 equiv of PMe_3_ directly in a J. Young NMR tube. The ^31^P{^1^H} NMR spectrum of **4** shows a triplet at 19.87 ppm for the carbon-bound PMe_3_ and a doublet at −17.94 ppm being flanked with ^183^W satellites (^1^*J*_WP_ = 277.8 Hz) for the tungsten-bound PMe_3_. In the ^13^C NMR spectrum, the methylene carbon resonates at 46.80 ppm (d, ^1^*J*_CP_ = 49.5 Hz). CO (q at 250.98 ppm) and W≡C (dt at 249.22 ppm) carbon atoms give signals of a similar shift and coupling pattern and could only be distinguished by heteronuclear multiple-bond correlation (HMBC) between the carbyne carbon and methylene protons. The W≡C resonance is similar to that of [W(CCH_3_)(PMe_3_)_4_Cl] (253.3 ppm)^32^ but shifted upfield compared to those of similar compounds like [W(CCH_2_Ph)(CO)_2_(dppe)Cl] (276.3 ppm),^33^ [Tp′(CO)_2_WCCH_2_W(CO)(C_2_Ph_2_)Tp] (312 ppm),^34^ and [Mo(CCH_2_B(C_6_F_5_)_3_)(dppe)_2_] (347.0 ppm).^35^ The byproduct 6-MePySCH_2_Cl was identified by ^1^H NMR and mass spectroscopy. When the reaction was carried out in CD_2_Cl_2_, the singlet for the methylene protons was absent. To a lesser extent, also bis((6-methylpyridin-2-yl)thio)methane [(6-MePyS)_2_CH_2_] was found, which was formed by the reaction of another 6-MePyS with the formerly generated 6-MePySCH_2_Cl. The W–C bond length of 1.793(4) Å in **4** confirms the triple-bond character, yet falls toward the shorter end of W≡C distances in recently published carbyne complexes (ca. 1.76–1.86 Å).^36−42^ The W1–C1–C2 angle of 179.4(4)° is almost perfectly linear, and the C1–C2 distance of 1.486(6) Å indicates a single bond.^43^ The P1–C2 bond [1.795(4) Å] is slightly longer than the other three P1–C bonds [1.761(5)–1.779(5) Å] but shorter than the remaining P–C bonds [1.807(6)–1.827(4) Å]. The W–S bond [2.6605(16) Å] is considerably longer than those in **1** [W1–S1 2.5834(12) Å; W1–S2 2.4073(12) Å], indicating a strong trans influence of the carbyne ligand.^44^

Treatment of a CH_2_Cl_2_ solution of **3** with 3 equiv of PMe_3_ led to an immediate color change from light yellow to dark green. After workup, the ethenyl complex [WO(CHCHPMe_3_)(PMe_3_)_2_(6-MePyS)]Cl (**5**; Scheme 3) was isolated in 90% yield as a black-green crystalline powder. Its structure was unambiguously identified by single-crystal X-ray diffraction analysis (Figure 2). Again, a nucleophilic attack at the coordinated C_2_H_2_ had occurred, forming a P–C bond. In contrast to **4**, however, **5** remains with a coordinated ethenyl ligand exhibiting a W–C single bond. Similar reactions were already performed with chromium(0)-bound C_2_H_2_; however, treatment with PMe_3_ predominantly led to an exchange with the coordinated C_2_H_2_.^45^ The ethenyl protons of **5** are clearly identified by ^1^H NMR spectroscopy with resonances at 11.42 and 4.26 ppm and coupling to each other with ^3^*J* = 17.5 Hz. Upon reaction of [WO(C_2_D_2_)(6-MePy_S_)_2_] (**3D**) with PMe_3_ in CD_2_Cl_2_, the ethenyl proton resonances are absent. The ^31^P{^1^H} NMR spectrum of **5** shows two rather broad singlets at 4.73 and −22.14 ppm, with the latter being flanked with ^183^W satellites. The ethenyl carbon bound to the tungsten center resonates at 222.99 ppm and thus exhibits a significant downfield shift compared to the literature. The other gives a doublet at 96.83 ppm and is thus considerably shifted upfield compared to similar compounds.^46,47^ The W–C distance of 2.068(3) Å is shorter than that in rare examples of tungsten ethenyl complexes like [WO_2_(CHCH_2_)(Tp′)] (2.136 Å)^46^ and [W(Cp)(CHCHC(CH_3_)_3_)(η^2^-C(O)NR^1^R^2^)(NO)] (2.161 Å),^47^ while the C1–C2 distance of 1.363(4) Å is slightly longer compared to the aforementioned compounds (1.305 and 1.332 Å). The W1–C1–C2 [135.8(2)°] and C1–C2–P1 [123.4(2)°] angle confirm a slight deviation from sp^2^ hybridization on the carbon atoms. In contrast to **4**, the P1–C2 bond [1.745(3) Å] is slightly shorter than the other three P1–C bonds [1.774(3)–1.796(3) Å].

In conclusion, we report the synthesis of tungsten acetylene complexes where intramolecular insertion of C_2_H_2_ into the W–N bond is prevented by steric adjustment at the ancillary ligand. This allowed the investigation of an intermolecular nucleophilic attack at the solely coordinated C_2_H_2_ using PMe_3_. Starting from the tungsten(II) complex **1**, a tungsten ethylidyne complex is formed; hence, the four-electron-donor C_2_H_2_ converts to the four-electron-donor carbyne when assuming retention of the metal oxidation state. When the carbyne is considered to be a six-electron donor, oxidation to tungsten(IV) is formally taking place. Regarding the oxido ligand as a six-electron donor, the two-electron-donor C_2_H_2_ in the tungsten(IV) complex **3** converts to a two-electron-donor ethenyl moiety. In both cases, the reactions proceed under preservation of the 18e^–^ character of the complexes. One 6-MePyS ligand is cleaved under reaction with CH_2_Cl_2_ to form 6-MePySCH_2_Cl and the counterion Cl^–^ for the cationic tungsten complexes; thus, the presence of CH_2_Cl_2_ is crucial. We assume that cleavage of the sulfur ligand relieves the charge at tungsten accumulated as a result of the nucleophilic attack. The attack of PMe_3_ on the tungsten(IV)-bound C_2_H_2_ to form an ethenyl ligand resembles the step in which the Inter2 intermediate of the proposed mechanism of AH is formed.

## Supporting Information Available

Experimental details, syntheses, crystallographic data, and NMR spectra of all compounds (PDF)

## Accession Codes

CCDC 2050311–2050316 contain the supplementary crystallographic data for this paper. These data can be obtained free of charge via www.ccdc.cam.ac.uk/data_request/cif, or by emailing data_request@ccdc.cam.ac.uk, or by contacting The Cambridge Crystallographic Data Centre, 12 Union Road, Cambridge CB2 1EZ, UK; fax: +44 1223 336033.

## Notes

^†^ Email: madeleine.ehweiner@uni-graz.at.

## Notes

^‡^ Email: lydia.peschel@uni-graz.at.

## Notes

^§^ Email: niklas.stix@edu.uni-graz.at.

## Notes

^⊥^ Email: miljan.corovic@uni-graz.at.

## Notes

^∥^ Email: ferdinand.belaj@uni-graz.at.
