# Supplementary material for: Bioinspired Nucleophilic Attack on a Tungsten-Bound Acetylene: Formation of Cationic Carbyne and Alkenyl Complexes
Source: Inorg Chem. 2021 Apr 14;60(12):8414–8. doi: 10.1021/acs.inorgchem.1c00643 (PMC8220502; doi:10.1021/acs.inorgchem.1c00643)
Supplement: Supplementary file 1 — ic1c00643_si_001.pdf [file ic1c00643_si_001.pdf]

Supporting Information

Bioinspired Nucleophilic Attack on a Tungsten-Bound Acetylene: Formation of Cationic Carbyne and Alkenyl Complexes

*Madeleine A. Ehweiner, Lydia M. Peschel, Niklas Stix, Miljan Z. Ćorović, Ferdinand Belaj, Nadia C. Mösch-Zanetti**

Institute of Chemistry – Inorganic Chemistry
University of Graz
Schubertstrasse 1, 8010 Graz

*Correspondence: nadia.moesch@uni-graz.at

Table of Contents

1 General Considerations S1
2 Syntheses S1
3 Crystal Structure Determination S6
4 NMR Spectra S17
5 References S37

1 General Considerations

All synthetic manipulations were performed under a nitrogen atmosphere using standard Schlenk and glovebox techniques. Solvents were purified via a Pure Solv Solvent Purification System. Chemicals were purchased from commercial sources and apart from acetylene, sodium hydride and pyridine *N*-oxide, all were used without further purification. Acetylene 2.6 was purified by bubbling it through water and conc. H_2SO_4 and subsequently dried by passing it through $CaCl_2$ and KOH. Pyridine *N*-oxide was purified by recrystallization from Et_2O and subsequent sublimation. NaH 60% in mineral oil was washed with pentane yielding pure NaH. Celite was dried at 100 °C prior to use, and silica gel was washed with Et_3N and subsequently dried in vacuo under heating. The metal precursor complex $[WBr_2(CO)_3(NCMe)_2]$[1] was synthesized according to established procedures. NMR spectra were recorded on a Bruker Avance III 300 MHz spectrometer at 22 °C. Chemical shifts δ are given in ppm. ^{1}H NMR spectra are referenced to residual protons in the solvent and ^{13}C NMR spectra to the deuterated solvent peak. Resonances in $^{31}P\{^{1}H\}$ NMR spectra were referenced to phosphoric acid as an external standard. The multiplicity of peaks is denoted as singlet (s), doublet (d), triplet (t), quadruplet (q), doublet of doublets (dd), doublet of triplets (dt), triplet of doublets (td), or multiplet (m). NMR solvents were stored over molecular sieves. Solid state IR spectra were measured on a Bruker ALPHA ATR-FT-IR spectrometer at a resolution of 2 cm^{-1}. The relative intensity of signals is declared as strong (s), medium (m), weak (w), and very weak (vw). Electron impact mass spectroscopy (EI-MS) measurements have been performed with an Agilent 5973 MSD mass spectrometer with a push rod. Elemental analyses (C, H, N, S) were performed at the Department of Inorganic Chemistry at the University of Technology in Graz and at the microanalytical laboratory of the University of Vienna. Values for elemental analyses are given as percentages.

2 Syntheses

The ligand 6-methylpyridine-2-thiol was synthesized from 6-methylpyridin-2-amine according to literature procedures.[2,3] It was then deprotonated with pure NaH in THF to yield Na(6-MePyS) in quantitative yield.[4]

In the solid state, complexes **1**, **3**, and **4** are stable at ambient conditions for a few days but ought to be stored and handled under a nitrogen atmosphere over a longer period of

time. Complex **2** is temperature-sensitive and slowly decomposes to **1** if not stored at –35 °C in a glove box. Compound **5** decomposes within 30 min at ambient conditions and ought to be stored in a glovebox, preferably at –35 °C.

[W(CO)(C_2H_2)(6-MePyS)$_2$] (1)

Na(6-MePyS) (927 mg, 6.30 mmol) was added portionwise to a stirred solution of [$WBr_2(CO)_3(NCMe)_2$] (1.53 g, 3.00 mmol) in 30 mL of CH_2Cl_2. After 40 min, the dark orange suspension was purged with acetylene for 30 min and subsequently stirred under an acetylene atmosphere for another 30 min. Thereafter, all volatiles were removed in vacuo. The purple solid was suspended in 100 mL of CH_2Cl_2, and the resulting suspension was filtrated through silica gel. The volume of the filtrate was reduced to 40 mL, whereupon 20 mL of heptane were added. Further evaporation to 10 mL gave a precipitate which was isolated by filtration, washed with 5 mL of pentane and eventually dried in vacuo to give [W(CO)(C_2H_2)(6-MePyS)$_2$] (1.25 g, 86%) as purple crystalline powder. ^{1}H NMR (CD_2Cl_2, 300 MHz): δ 13.77 (s, 1H, C≡CH), 12.50 (s, 1H, C≡CH), 7.55 (t, *J* = 7.8 Hz, 1H, pyH-*p*), 7.04 (t, *J* = 7.8 Hz, 1H, pyH-*p*), 6.92 (d, *J* = 7.7 Hz, 1H, pyH-*m*), 6.71 (d, *J* = 7.8 Hz, 1H, pyH-*m*), 6.59 (m, 2H, pyH-*m*), 1.91 (s, 3H, CH_3), 1.22 (s, 3H, CH_3) ppm. ^{13}C NMR (CD_2Cl_2, 75 MHz): δ 237.09 (CO), 205.73 (C≡CH), 204.14 (C≡CH), 178.36 (pyC-*o*), 170.65 (pyC-*o*), 158.31 (pyC-*o*), 154.67 (pyC-*o*), 138.77 (pyC-*p*), 135.97 (pyC-*p*), 124.56 (pyC-*m*), 124.40 (pyC-*m*), 120.25 (pyC-*m*), 118.67 (pyC-*m*), 26.88 (CH_3), 22.08 (CH_3) ppm. IR (cm^{-1}): 3102 (w), 2991 (w), 1891 (s, C≡O), 1854 (s, C≡O), 1583 (m), 1550 (m), 1510 (m), 1447 (m), 1431 (m), 1170 (m), 774 (m). EI-MS (70 eV) *m/z*: M^+ 486.0, $[M – CO]^+$ 458.1, $[M – CO – C_2H_2]^+$ 431.0. Anal. Calcd. for $C_{15}H_{14}N_2OS_2W$: C, 37.05; H, 2.90; N, 5.76; S, 13.19. Found: C, 36.98; H, 2.91; N, 5.88; S, 13.17.

[W(CO)(C_2D_2)(6-MePyS)$_2$] (1D)

Same procedure as for **1**. Deuterated acetylene was prepared by addition of D_2O to CaC_2 and subsequently dried by passing it through a glass tube packed with $CaCl_2$. ^{1}H NMR ($CDCl_3$, 300 MHz): δ 7.51 (t, *J* = 7.8 Hz, 1H, pyH-*p*), 7.00 (t, *J* = 7.8 Hz, 1H, pyH-*p*), 6.88 (d, *J* = 7.6 Hz, 1H, pyH-*m*), 6.72 (d, *J* = 8.0 Hz, 1H, pyH-*m*), 6.61 (d, *J* = 8.1 Hz, 1H, pyH-*m*), 6.56 (d, *J* = 7.5 Hz, 1H, pyH-*m*), 1.91 (s, 3H, CH_3), 1.21 (s, 3H, CH_3) ppm.

[W(CO)(C_2H_2)(HCCH-6-MePyS)(6-MePyS)] (2)

Na(6-MePyS) (309 mg, 2.10 mmol) was added portionwise to a stirred solution of [$WBr_2(CO)_3(NCMe)_2$] (510 mg, 1.00 mmol) in 16 mL of CH_2Cl_2 and 16 mL of MeCN. After 45 min, the yellow-orange suspension was purged with acetylene for 1 h and stirred under an acetylene atmosphere. This procedure was repeated twice in appropriate intervals. After 24 h of stirring, the reaction mixture was evaporated to dryness. The reddish solid was suspended in 40 mL of CH_2Cl_2, and the resulting suspension was filtrated through silica gel. Subsequently, the deep pink filtrate was evaporated to dryness. Repeated recrystallization of the respective crystals from CH_2Cl_2/heptane at –25 °C gave [W(CO)(C_2H_2)(HCCH-6-MePyS)(6-MePyS)] (125 mg, 24%) as orange-red crystals. ^{1}H NMR (CD_2Cl_2, 300 MHz): δ 12.90 (s, 1H, HC≡C), 12.03 (s, 1H, HC≡C), 7.80 (d, *J* = 8.5 Hz, 1H, pyH-*m*), 7.61 (d, ^3J = 10.9 Hz, 1H, η^1-C_2H_2), 7.38 (t, *J* = 7.8 Hz, 1H, pyH-*p*), 7.08 (dd, 1H, pyH-*p*), 6.89 (d, ^3J = 10.9 Hz, 1H, η^1-C_2H_2), 6.82 (d, *J* = 7.1 Hz, 1H, pyH-*m*), 6.74 (d, *J* = 7.6 Hz, 1H, pyH-*m*), 6.60 (d, *J* = 8.0 Hz, 1H, pyH-*m*), 2.51 (s, 3H, CH_3), 2.45 (s, 3H, CH_3) ppm. ^{13}C NMR (CD_2Cl_2, 75 MHz): δ 231.31 (CO), 193.95 (C≡CH), 191.07 (C≡CH), 178.81 (pyC-*o*), 173.22 (η^1-C_2H_2), 160.85 (pyC-*o*), 159.02 (pyC-*o*), 147.09 (pyC-*o*), 137.60 (pyC-*p*), 132.01 (pyC-*m*), 131.72 (pyC-*p*), 127.73 (η^1-C_2H_2), 124.69 (pyC-*m*), 119.79 (pyC-*m*), 118.03 (pyC-*m*), 23.92 (CH_3), 23.27 (CH_3) ppm. IR (cm^{-1}): 1897 (s, C≡O), 1857 (w), 1617 (w), 1585 (w), 1555 (m), 1514 (m), 1430 (m), 1370 (m), 1172 (m), 1008 (m), 881 (m), 769 (s). Anal. Calcd. for $C_{17}H_{16}N_2OS_2W$: C, 39.86; H, 3.15; N, 5.47; S, 12.52. Found: C, 39.83; H, 3.08; N, 5.58; S, 12.68.

[WO(C_2H_2)(6-MePyS)$_2$] (3)

A solution of [W(CO)(C_2H_2)(6-MePyS)$_2$] (729 mg, 1.50 mmol) and pyridine *N*-oxide (164 mg, 1.72 mmol) in 30 mL of CH_2Cl_2 was stirred for 20 h. After evaporation to dryness, the beige solid was suspended in 50 mL of CH_2Cl_2, and the resulting mixture was filtrated through Celite yielding a yellow filtrate. The volume of the filtrate was reduced to 20 mL before 15 mL of MeCN was added. Further evaporation gave crystals which were then isolated by filtration, washed with 2 mL of MeCN and 2x 2 mL of Et_2O, and eventually dried in vacuo to yield [WO(C_2H_2)(6-MePyS)$_2$] (600 mg, 84%) as light yellow crystalline solid. ^{1}H NMR (CD_2Cl_2, 300 MHz): δ 11.23 (s(d), 1H, $^2J_{WH}$ = 10.1 Hz, C≡CH), 10.99 (s(d), 1H, $^2J_{WH}$ = 11.5 Hz, C≡CH), 7.59 (t, *J* = 7.9 Hz, 1H, pyH-*p*), 7.45 (t, *J* = 7.8 Hz, 1H, pyH-*p*), 7.02 (d, *J* = 7.5 Hz, 1H, pyH-*m*), 6.96 (m, 2H, pyH-*m*), 6.77 (d, *J* = 8.1 Hz, 1H, pyH-*m*), 2.61 (s, 3H, CH_3), 2.06 (s, 3H, CH_3) ppm. ^{13}C NMR (CD_2Cl_2,

75 MHz): δ 175.22 (pyC-*o*), 173.05 (pyC-*o*), 159.69 (s(d), J_{WC} = 30.2 Hz, C≡CH), 159.05 (s(d), J_{WC} = 31.6 Hz, C≡CH), 158.12 (pyC-*o*), 155.84 (pyC-*o*), 139.42 (pyC-*p*), 137.69 (pyC-*p*), 126.11 (pyC-*m*), 124.80 (pyC-*m*), 121.11 (pyC-*m*), 119.15 (pyC-*m*), 25.39 (CH_3), 21.20 (CH_3) ppm. IR (cm^{-1}): 1589 (m), 1556 (m), 1433 (m), 1177 (m), 963 (w), 924 (s, W=O), 775 (s), 695 (s). EI-MS (70 eV) *m/z*: M^+ 474.0, $[M – C_2H_2]^+$ 448.0. Anal. Calcd. for $C_{14}H_{14}N_2OS_2W$: C, 35.46; H, 2.98; N, 5.91; S, 13.52. Found: C, 35.68; H, 2.89; N, 6.06; S, 13.54.

[WO(C_2D_2)(6-MePyS)$_2$] (3D)

Same procedure as for **3**. Deuterated acetylene was prepared by addition of D_2O to CaC_2 and subsequently dried by passing it through a glass tube packed with $CaCl_2$. ^{1}H NMR ($CDCl_3$, 300 MHz): 7.54 (t, J = 7.9 Hz, 1H, pyH-*p*), 7.42 (t, J = 7.8 Hz, 1H, pyH-*p*), 7.00–6.95 (m, 2H, pyH-*m*), 6.91 (d, J = 7.7 Hz, 1H, pyH-*m*), 6.80 (d, J = 8.1 Hz, 1H, pyH-*m*), 2.64 (s, 3H, CH_3), 2.07 (s, 3H, CH_3) ppm.

[W(CO)(CCH_2PMe_3)(PMe_3)$_2$(6-MePyS)]Cl (4)

A solution of [W(CO)(C_2H_2)(6-MePyS)$_2$] (194 mg, 0.40 mmol) and PMe_3 (152 µL, 1.48 mmol) in 7 mL of CH_2Cl_2 was stirred for 4 h. After evaporation to dryness, the dark orange solid was dissolved in 12 mL of CH_2Cl_2 and 11 mL of toluene. Then, the volume was reduced to approx. 10 mL, and the resulting solid was isolated by cannulation, washed with 3x 10 mL of Et_2O and 3x 10 mL of pentane and eventually dried in vacuo to yield [W(CO)(CCH_2PMe_3)(PMe_3)$_2$(6-MePyS)]Cl (125 mg, 50%) as orange powder. ^{1}H NMR (CD_2Cl_2, 300 MHz): δ 7.14 (t, 1H, pyH-*p*), 6.78 (d, J = 7.5 Hz, 1H, pyH-*m*), 6.49 (d, J = 8.0 Hz, 1H, pyH-*m*), 3.81 (dt, J = 19.4, 5.0 Hz, 2H, CH_2), 2.33 (s, 3H, CH_3), 2.12 (d, $^2J_{HP}$ = 14.3 Hz, 9H, PCH_3), 1.42 (t, $^2J_{HP}$ = 3.6 Hz, 18H, $WPCH_3$) ppm. ^{13}C NMR (CD_2Cl_2, 75 MHz): δ 250.98 (q, J = 4.7 Hz, CO), 249.22 (dt, J = 11.5, 13.7 Hz, W≡C), 175.52 (td, J_{CP} = 1.5, 3.3 Hz, pyC-o), 155.95 (d, J_{CP} = 1.0 Hz, pyC-*o*), 135.81 (t, J_{CP} = 2.0 Hz, pyC-*p*), 126.49 (pyC-*m*), 117.36 (pyC-*m*), 46.80 (d, $^1J_{CP}$ = 49.5 Hz, CH_2), 24.94 (CH_3), 18.55 (t, $^1J_{CP}$ = 13.8 Hz, 6C, $WPCH_3$), 9.51 (d, $^1J_{CP}$ = 54.0 Hz, 3C, PCH_3) ppm. ^{31}P{^{1}H} NMR (CD_2Cl_2, 121 MHz): δ 19.87 (t, J = 5.8 Hz, PMe_3), –17.94 (d(dd), J = 5.8, 278 Hz, $WPMe_3$) ppm. IR (cm^{-1}): 2966 (w), 2902 (w), 2861 (w), 2819 (w), 1847 (s, C≡O), 1581 (w), 1551 (w), 1428 (w), 1280 (w), 1169 (w), 1153 (w), 970 (m), 944 (s), 776 (m), 731 (m), 670 (m). Anal. Calcd. for $C_{18}H_{35}NOP_3SClW$: C, 34.55; H, 5.64; N, 2.24; S, 5.12. Found: C, 34.27; H, 5.97; N, 2.06; S, 4.60.

[WO(CHCHPMe$_3$)(PMe$_3$)$_2$(6-MePyS)]Cl (5)

A solution of [WO(C_2H_2)(6-MePyS)$_2$] (237 mg, 0.50 mmol) and PMe$_3$ (196 µL, 1.90 mmol) in 10 mL of CH_2Cl_2 was stirred for 7 h. After evaporation to dryness, the dark green solid was dissolved in 12 mL of CH_2Cl_2 and 12 mL of toluene. Then, the volume was reduced to 8 mL, and the resulting solid was isolated by cannulation, washed with 2x 5 mL of Et_2O and 5 mL of pentane and eventually dried in vacuo to yield [WO(CHCHPMe$_3$)(PMe$_3$)$_2$(6-MePyS)]Cl (275 mg, 90%) as black-green crystalline powder. ^{1}H NMR (CD_2Cl_2, 300 MHz): δ 11.42 (dd, *J* = 17.4, 36.7 Hz, 1H, CH), 7.13 (t, *J* = 7.8 Hz, 1H, pyH-*p*), 6.95 (d, *J* = 7.5 Hz, 1H, pyH-*m*), 6.56 (d, *J* = 8.0 Hz, 1H, pyH-*m*), 4.26 (dd, *J* = 17.7, 35.7 Hz, 1H, CH), 2.52 (s, 3H, CH$_3$), 1.94 (d, $^2J_{PH}$ = 13.5 Hz, 9H, PCH$_3$), 1.40 (t, $^2J_{PH}$ = 4.2 Hz, 18H, WPCH$_3$) ppm. ^{13}C NMR (CD_2Cl_2, 75 MHz): δ 222.99 (WCH), 165.80 (pyC-*o*), 154.67 (pyC-*o*), 135.31 (pyC-*p*), 125.39 (pyC-*m*), 118.57 (pyC-*m*), 96.81 (d, $^1J_{CP}$ = 79.5 Hz, CH), 24.83 (CH$_3$), 14.28 (t, $^1J_{CP}$ = 15.1 Hz, 6C, WPCH$_3$), 13.16 (d, $^1J_{CP}$ = 57.9 Hz, 3C, PCH$_3$) ppm. ^{31}P{^{1}H} NMR (CD_2Cl_2, 121 MHz): δ 4.73 (s, PMe$_3$), –22.14 (s(d), *J* = 339.0 Hz, WPMe$_3$) ppm. IR (cm^{-1}): 2968 (w), 2898 (w), 1580 (w), 1546 (w), 1432 (m), 1408 (m), 1281 (m), 1257 (m), 1172 (m), 1147 (m), 981 (m), 941 (s, W=O), 898 (m), 862 (m), 761 (m), 730 (m), 670 (m), 585 (w). Anal. Calcd. for $C_{17}H_{35}NOP_3SClW$: C, 33.27; H, 5.75; N, 2.28; S, 5.22. Found: C, 32.87; H, 5.73; N, 2.28; S, 5.07.

NMR data of 2-((chloromethyl)thio)-6-methylpyridine

^{1}H NMR (CD_2Cl_2, 300 MHz): δ 7.49 (t, *J* = 7.7 Hz, 1H, pyH-*p*), 7.08 (d, *J* = 7.9 Hz, 1H, pyH-*m*), 6.98 (d, *J* = 7.77 Hz, 1H, pyH-*m*), 5.41 (s, 2H, CH$_2$), 2.54 (s, 3H, CH$_3$) ppm. EI-MS (70 eV) *m/z*: [M – Cl]$^+$ 138.0, [C_6H_6N]$^+$ 92.0, [CH_2SCl]$^+$ 81.0,

NMR data of Bis((6-methylpyridin-2-yl)thio)methane

^{1}H NMR (CD_2Cl_2, 300 MHz): δ 7.41 (t, *J* = 7.7 Hz, 2H, pyH-*p*), 7.00 (d, *J* = 8.4 Hz, 2H, pyH-*m*), 6.90 (d, *J* = 7.5 Hz, 2H, pyH-*m*), 5.07 (s, 2H, CH$_2$), 2.52 (s, 6H, CH$_3$) ppm. EI-MS (70 eV) *m/z*: M$^+$ 262.0, [$C_7H_8NS_2$]$^+$ 170.0, [C_6H_6N]$^+$ 92.0.

3 Crystal Structure Determination

General. All the single crystal measurements were performed on a Bruker APEX-II CCD diffractometer at 100 K using Mo K_{α} radiation with a wavelength of 0.71073 Å from an Incoatec microfocus sealed tube equipped with a multilayer monochromator. Absorption corrections were performed semiempirically from equivalents. Molecular structures were solved by direct methods (SHELXS-97)[5] and refined by full-matrix least-squares techniques against F^2 (SHELXL-2014/6)[6]. A weighting scheme of $w = 1/[\sigma^2(F_o^2)+(aP)^2+bP]$ where $P = (F_o^2+2F_c^2)/3$ was used. The non-hydrogen atoms of the metal complexes and of the corresponding anion Cl1 in **4** were refined with anisotropic displacement parameters without any constraints. The positions of the H atoms of the of the ethyne ligands, of the ethenidyl group in **2**, and of the ethenyl group in **5** were taken from difference Fourier maps, the C–H distances were fixed to 0.95 Å, and these H atoms were refined with isotropic displacement parameters without any constraints to the bond angles. The H atoms of the CH_2 group in **4** were refined with a common isotropic displacement parameter and idealized geometry with approx. tetrahedral angles and C–H distances of 0.99 Å. The H atoms of the pyridine rings were put at the external bisectors of the C–C–C angles at C–H distances of 0.95 Å, and a common isotropic displacement parameter was refined for the H atoms of the same ring. Except for compound **4** (see below), the H atoms of the methyl groups were refined with common isotropic displacement parameters for the H atoms of the same group and idealized geometries with tetrahedral angles, enabling rotation around the C–X bonds, and C–H distances of 0.98 Å. Crystal data, data collection parameters and structure refinement details are given in Tables S1–S3. Further refinement information, structure and bonding parameters, SHELXL .res and .hkl files are given in the deposited CIF file which is available free of charge from The Cambridge Crystallographic Data Centre (CCDC 2050311–2050316).

Crystal Structure Determination of 4. The tetramethylphosphonium cation was disordered over two orientations around a two-fold rotation axis and the corresponding chloride anion was disordered over four orientations near this axis. In this disordered part the P–C bonds were restrained to have the same value resulting in 1.799(7) Å, the cations were refined as rigid bodies, and the same anisotropic displacement parameters were used for the disordered Cl atom. For the latter, some restraints were also given to the anisotropic displacement parameters and the P atoms of the cations were restrained to

have similar U_{ij} components. Besides the described ion pairs there are large regions containing disordered solvent molecules. Dichloromethane and n-heptane were used in the crystallization process. Since it was not possible to describe the refined peaks of residual electron density as partially occupied individual solvent molecules, the diffuse electron density in these regions was removed by the SQUEEZE routine of the PLATON program[7]. The solvent contributions to the calculated structure factors for each reflection hkl is included in the cif file. The SQUEEZE routine resulted in two void volumes of 757 $Å^3$ each per unit cell (20.7% of the unit cell) with an electron count of 598 per unit cell. The solvent molecules were not included in the reported chemical formula, molecular weight, density, and F000. The R value R1 after the use of SQUEEZE was almost the same as by description of the electron density by 29 partially occupied C atoms. The use of SQUEEZE decreased the R value R1 from 0.0948 to 0.0444. The H atoms of the methyl groups of the metal cation were refined with common isotropic displacement parameters for the H atoms of the same trimethylphosphine, trimethylphosphonium, or methylpyridine group, resp., and idealized geometries with tetrahedral angles, enabling rotations around the C–C or P–C bonds, resp., and C–H distances of 0.98 Å. The H atoms of the tetramethylphosphonium cation were included with their isotropic displacement parameters fixed to 1.2 times U_{eq} of the C atom they are bonded to and idealized geometries with tetrahedral angles, staggered conformations, and C–H distances of 0.98 Å. The largest peak in a final difference Fourier map (1.94 $eÅ^{-3}$) was found in a distance of 1.82 Å of the disordered atom Cl5 and should have been removed by the SQUEEZE routine. The next largest peaks (0.96–1.57 $eÅ^{-3}$) were in the vicinity (0.41–0.83 Å) of the W atom.

Crystallographic Data

Table S1. Crystallographic data and structure refinement for [$W(CO)_3(6\text{-}MePyS)_2$] and **1**.

**Crystal data**	**[$W(CO)_3(6\text{-}MePyS)_2$]**	**[$W(CO)(C_2H_2)(6\text{-}MePyS)_2$] (1)**
CIF data code	MC2A	LP394B
Empirical formula	$C_{15}H_{12}N_2O_3S_2W$	$C_{15}H_{14}N_2OS_2W$
Formula weight	516.24	486.25
Crystal description	needle, red	plate, red
Crystal size	0.31 x 0.13 x 0.06 mm	0.25 x 0.16 x 0.05mm
Crystal system	monoclinic	orthorhombic
Space group	P 2_1/n	P 2_1 2_1 2_1
a	8.0079(4) Å	7.1571(9) Å
b	17.2211(8) Å	7.9622(10) Å
c	11.8493(6) Å	27.001(4) Å
β	99.268(2)°	
Volume	1612.74(14) $Å^3$	1538.7(3) $Å^3$
Z	4	4
Calc. density	2.126 Mg/m^3	2.099 Mg/m^3
F(000)	984	928
Linear absorption coefficient μ	7.436 mm^{-1}	7.777 mm^{-1}
Max. and min. transmission	1.000 and 0.455	1.000 and 0.595
Unit cell determination	2.84° < Θ < 40.76°	2.67° < Θ < 35.62°
Reflections used	9847	9077
**Data collection**		
Θ range for data collection	2.11 to 40.00°	2.67 to 35.00°
Reflections collected/ unique	52144 / 9993	19551 / 6508
Significant unique reflections	8630 with I > 2σ(I)	5957 with I > 2σ(I)
R(int), R(sigma)	0.0521, 0.0372	0.0373, 0.0481
Completeness to Θ_{max}	100.0%	99.6%
**Refinement**		
Data/ parameters/ restraints	9993 / 214 / 0	6508 / 203 / 2
Goodness-of-fit on F^2	1.031	1.086
Final R indices [I > 2σ(I)]	R1 = 0.0243, wR2 = 0.0560	R1 = 0.0303, wR2 = 0.0587
R indices (all data)	R1 = 0.0311, wR2 = 0.0588	R1 = 0.0348, wR2 = 0.0608
Weighting scheme param. a, b	0.0232, 0.8201	0.0102, 3.1401
Largest Δ/σ in last cycle	0.003	0.002
Largest diff. peak and hole	3.148 and -1.339 e/$Å^3$	1.601 and -1.350 e/$Å^3$
**CCDC no.**	2050311	2050312

Table S2. Crystallographic data and structure refinement for **2** and **3**.

**Crystal data**	**[W(CO)(C_2H_2)(HCCH-6-MePyS)(6-MePyS)] (2)**	**[WO(C_2H_2)(6-MePyS)$_2$] (3)**
CIF data code	ME176	LP396
Empirical formula	$C_{17}H_{16}N_2OS_2W$	$C_{14}H_{14}N_2OS_2W$
Formula weight	512.29	474.24
Crystal description	plate, orange	plate, yellow
Crystal size	0.29 x 0.18 x 0.05 mm	0.25 x 0.18 x 0.11 mm
Crystal system	triclinic	monoclinic
Space group	P -1	P 2_1/c
a	8.0503(4) Å	8.6692(9) Å
b	8.5121(4) Å	25.298(3) Å
c	13.4133(6) Å	7.5044(8) Å
α	99.4523(18)°	
β	90.149(2)°	114.615(3)°
γ	112.2705(16)°	
Volume	836.91(7) Å^3	1496.3(3) Å^3
Z	2	4
Calc. density	2.033 Mg/m^3	2.105 Mg/m^3
F(000)	492	904
Linear absorption coefficient μ	7.155 mm^{-1}	7.995 mm^{-1}
Max. and min. transmission	1.000 and 0.525	1.000 and 0.696
Unit cell determination	2.74° < Θ < 40.98°	2.71° < Θ < 40.88°
Reflections used	9965	9936
**Data collection**		
Θ range for data collection	2.63 to 40.00°	2.58 to 40.00°
Reflections collected/ unique	23480 / 10378	26940 / 9264
Significant unique reflections	9329 with I > 2σ(I)	8078 with I > 2σ(I)
R(int), R(sigma)	0.0397, 0.0536	0.0300, 0.0357
Completeness to Θ_{max}	99.9%	99.9%
**Refinement**		
Data/ parameters/ restraints	10378 / 228 / 4	9264 / 194 / 2
Goodness-of-fit on F^2	1.041	1.031
Final R indices [I > 2σ(I)]	R1 = 0.0291, wR2 = 0.0694	R1 = 0.0232, wR2 = 0.0461
R indices (all data)	R1 = 0.0354, wR2 = 0.0724	R1 = 0.0292, wR2 = 0.0475
Weighting scheme param. a, b	0.0159, 1.5275	0.0146, 0.6335
Largest Δ/σ in last cycle	0.002	0.007
Largest diff. peak and hole	2.299 and -1.825 e/Å^3	2.063 and -1.402 e/Å^3
**CCDC no.**	2050314	2050313

Table S3. Crystallographic data and structure refinement for **4** and **5**.

**Crystal data**	**[W(CO)(CCH$_2$PMe$_3$)(PMe$_3$)$_2$ (6-MePyS)]Cl (4)**	**[WO(CHCHPMe$_3$)(PMe$_3$)$_2$ (6-MePyS)]Cl (5)**
CIF data code	ME254	ME263
Empirical formula	2($C_{18}H_{35}NOP_3SW^+Cl^-$) · $(CH_3)_4P^+Cl^-$	$C_{17}H_{35}NOP_3SW^+Cl^-$
Formula weight	1378.03	613.73
Crystal description	block, orange	plate, red
Crystal size	0.37 x 0.23 x 0.22 mm	0.16 x 0.16 x 0.05 mm
Crystal system	monoclinic	orthorhombic
Space group	C 2/c	P b c a
a	33.707(2) Å	11.7141(5) Å
b	12.2501(8) Å	18.9559(7) Å
c	18.7867(12) Å	23.0603(9) Å
β	109.463(3)°	
Volume	7314.1(8) Å^3	5120.6(4) Å^3
Z	4	8
Calc. density	1.251 Mg/m^3	1.592 Mg/m^3
F(000)	2752	2432
Linear absorption coefficient μ	3.488 mm^{-1}	4.892 mm^{-1}
Max. and min. transmission	0.746 and 0.309	0.746 and 0.424
Unit cell determination	2.51° < Θ < 29.78°	2.90° < Θ < 29.97°
Reflections used	9229	9749
**Data collection**		
Θ range for data collection	1.78 to 30.00°	1.77 to 30.00°
Reflections collected/ unique	141906 / 10671	126328 / 7468
Significant unique reflections	6017 with I > 2σ(I)	5700 with I > 2σ(I)
R(int), R(sigma)	0.0671, 0.0806	0.1018, 0.0564
Completeness to Θ_{max}	100.0%	99.9%
**Refinement**		
Data/ parameters/ restraints	10671 / 304 / 18	7468 / 255 / 2
Goodness-of-fit on F^2	1.018	1.064
Final R indices [I > 2σ(I)]	R1 = 0.0444, wR2 = 0.1062	R1 = 0.0355, wR2 = 0.0542
R indices (all data)	R1 = 0.0917, wR2 = 0.1212	R1 = 0.0603, wR2 = 0.0610
Weighting scheme param. a, b	0.0486, 0.0000	0.0000, 4.0792
Largest Δ/σ in last cycle	0.002	0.002
Largest diff. peak and hole	1.935 and -1.447 e/Å^3	1.613 and -1.194 e/Å^3
**CCDC no.**	2050315	2050316

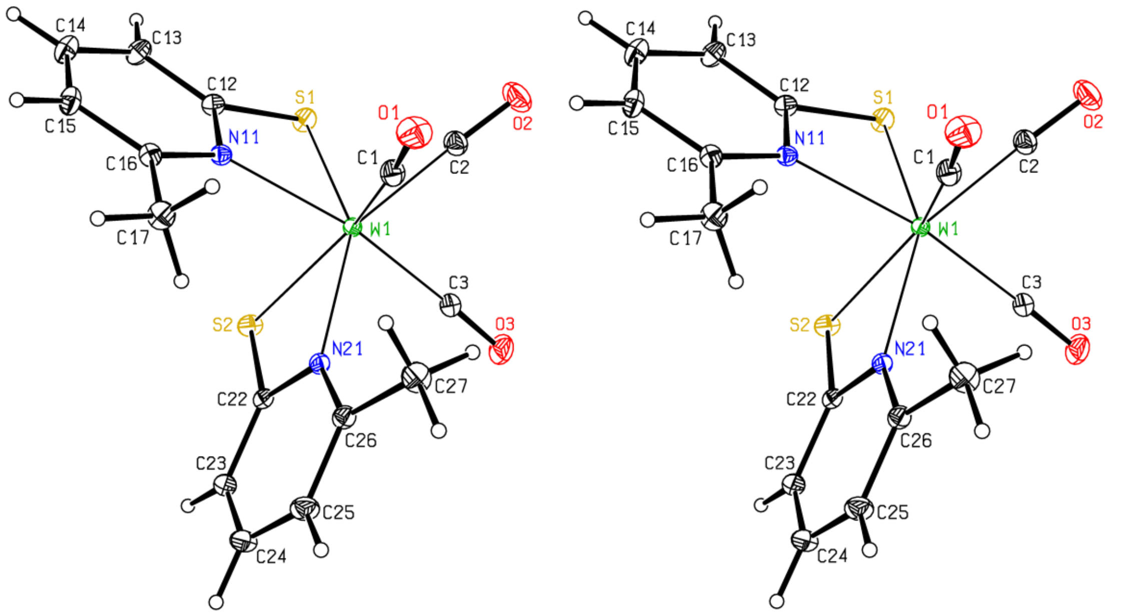

Figure S1. Stereoscopic ORTEP[8] plot of $[W(CO)_3(6\text{-}MePyS)_2]$ showing the atomic numbering scheme. The probability ellipsoids are drawn at the 50% probability level. The H atoms are drawn with arbitrary radii.

Table S4. Selected bond lengths (Å) and angles (°) for $[W(CO)_3(6\text{-}MePyS)_2]$.

W1–C1	2.0024(17)	C3–W1–N11	167.10(6)
W1–C2	1.9859(17)	N21–W1–S1	144.83(4)
W1–C3	2.0141(18)	C2–W1–2)	141.15(5)
W1–N11	2.2282(14)	C1–W1–S2	148.46(5)
W1–N21	2.2447(14)	S1–W1–S2	81.633(15)
W1–S1	2.4967(4)	N11–W1–N21	98.15(5)
W1–S2	2.5352(4)	C12–S1–W1	82.58(6)
C1–O1	1.149(2)	C22–S2–W1	81.39(6)
C2–O2	1.155(2)	C16–N11–C12	120.10(14)
C3–O3	1.143(2)	C16–N11–W1	137.27(11)
S1–C12	1.7326(17)	C12–N11–W1	102.46(10)
S2–C22	1.7395(17)	C26–N21–C22	119.98(14)
		C26–N21–W1	138.23(11)
		C22–N21–W1	101.72(10)

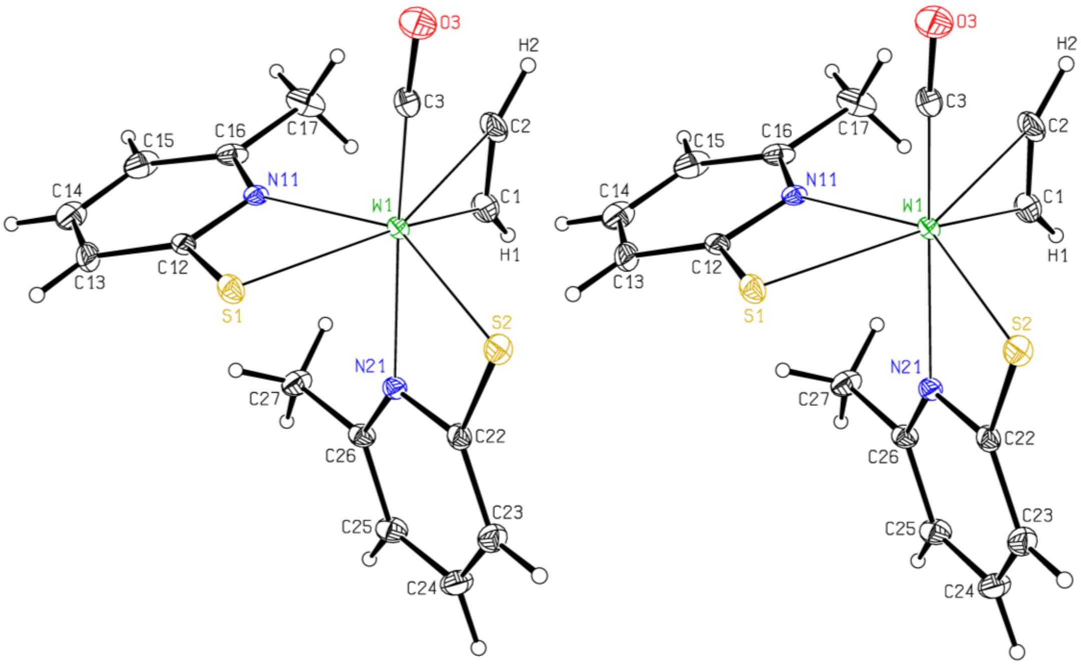

Figure S2. Stereoscopic ORTEP[8] plot of **1** showing the atomic numbering scheme. The probability ellipsoids are drawn at the 50% probability level. The H atoms are drawn with arbitrary radii.

Table S5. Selected bond lengths (Å) and angles (°) for **1**.

W1–C1	2.022(5)	N11–W1–S2	146.57(12)
W1–C2	2.055(3)	C2–C1–H1	141.4(12)
W1–C3	1.958(5)	C1–C2–H2	137.6(9)
W1–N11	2.197(3)	O3–C3–W1	177.0(4)
W1–N21	2.259(4)	C12–S1–W1	79.99(16)
W1–S1	2.5834(12)	C12–N11–C16	119.3(4)
W1–S2	2.4073(12)	C12–N11–W1	103.9(3)
C1–C2	1.306(7)	C16–N11–W1	136.2(4)
C3–O3	1.167(6)	C22–S2–W1	83.78(16)
S1–C12	1.739(6)	C26–N21–C22	120.3(4)
S2–C22	1.760(6)	C26–N21–W1	139.8(3)
		C22–N21–W1	99.4(3)
C1–W1–S1	156.74(14)		
C2–W1–S1	156.04(15)	C1–C2–W1–C3	179.7(5)
C3–W1–N21	162.54(18)	C2–C1–W1–C3	–0.3(7)

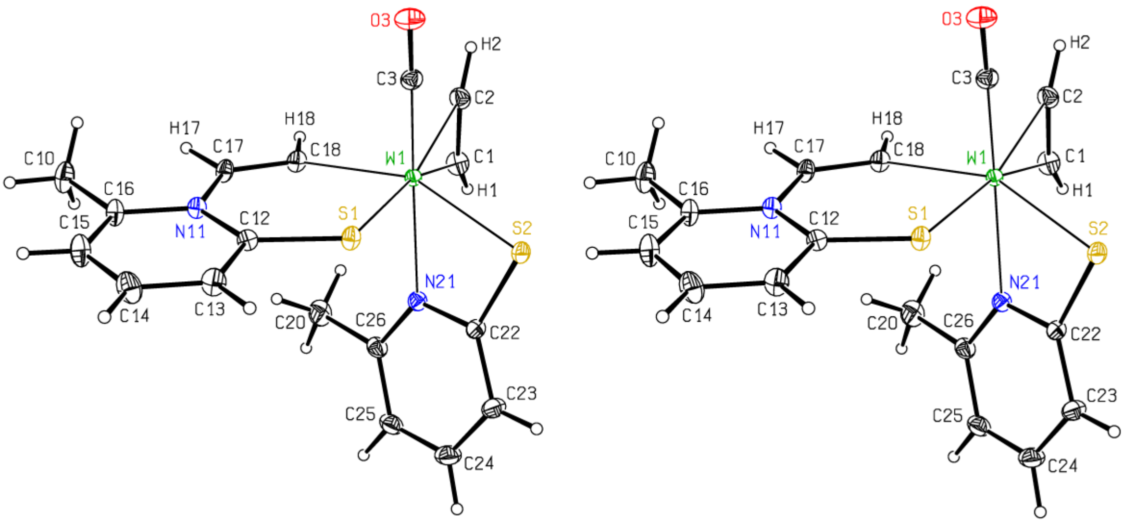

Figure S3. Stereoscopic ORTEP[8] plot of **2** showing the atomic numbering scheme. The probability ellipsoids are drawn at the 50% probability level. The H atoms are drawn with arbitrary radii.

Table S6. Selected bond lengths (Å) and angles (°) for **2**.

W1–C1	2.047(2)	C18–W1–S2	149.86(6)
W1–C2	2.065(2)	C2–C1–H1	145.7(8)
W1–C3	1.974(2)	C1–C2–H2	143.1(13)
W1–C18	2.103(2)	O3–C3–W1	179.2(2)
W1–N21	2.2483(19)	C12–S1–W1	118.43(8)
W1–S1	2.5484(6)	C18–C17–N11	128.80(18)
W1–S2	2.4794(6)	C18–C17–H17	116.8(16)
C1–C2	1.310(3)	N11–C17–H17	114.2(16)
C3–O3	1.168(3)	C17–C18–W1	143.87(15)
S1–C12	1.722(2)	C17–C18–H18	114.2(15)
N11–C17	1.430(3)	W1–C18–H18	101.8(16)
C17–C18	1.349(3)	C22–S2–W1	82.63(7)
S2–C22	1.755(2)	C26–N21–C22	120.6(2)
		C26–N21–W1	137.48(16)
C1–W1–S1	163.68(7)	C22–N21–W1	101.60(14)
C2–W1–S1	159.10(7)		
C3–W1–N21	161.36(9)		

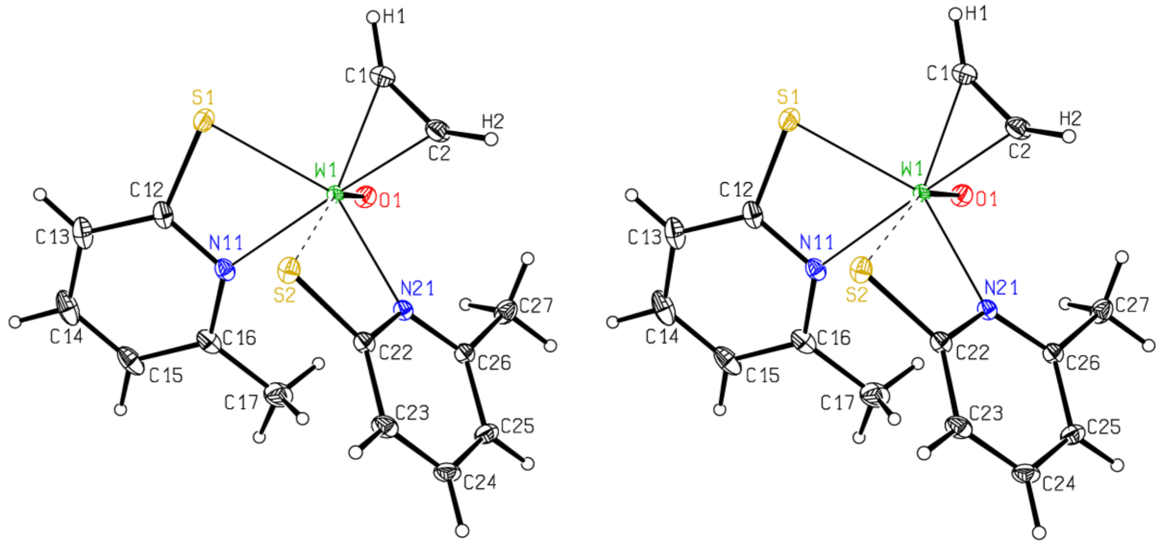

Figure S4. Stereoscopic ORTEP[8] plot of **3** showing the atomic numbering scheme. The probability ellipsoids are drawn at the 50% probability level. The H atoms are drawn with arbitrary radii.

Table S7. Selected bond lengths (Å) and angles (°) for **3**.

W1–O1	1.7153(13)	C1–C2–H2	145.4(6)
W1–C1	2.0693(15)	C12–S1–W1	84.26(6)
W1–C2	2.1027(15)	C16–N11–C12	119.84(14)
W1–N11	2.2637(13)	C16–N11–W1	139.68(11)
W1–N21	2.1971(13)	C12–N11–W1	100.32(10)
W1–S1	2.4177(4)	C22–S2–W1	79.09(6)
W1–S2	2.6367(5)	C22–N21–C26	120.95(13)
C1–C2	1.279(2)	C22–N21–W1	105.03(9)
S1–C12	1.7551(18)	C26–N21–W1	133.80(11)
S2–C22	1.7253(16)		
		C1–C2–W1–O1	89.66(12)
C1–W1–N11	147.30(6)	C2–C1–W1–O1	–98.29(12)
C2–W1–N11	168.27(6)	C1–C2–W1–N21	–177.07(12)
N21–W1–S1	145.05(4)	C2–C1–W1–N21	3.38(13)
O1–W1–S2	152.59(4)	C1–C2–W1–S1	–28.05(13)
C2–C1–H1	142.8(8)	C2–C1–W1–S1	154.15(12)

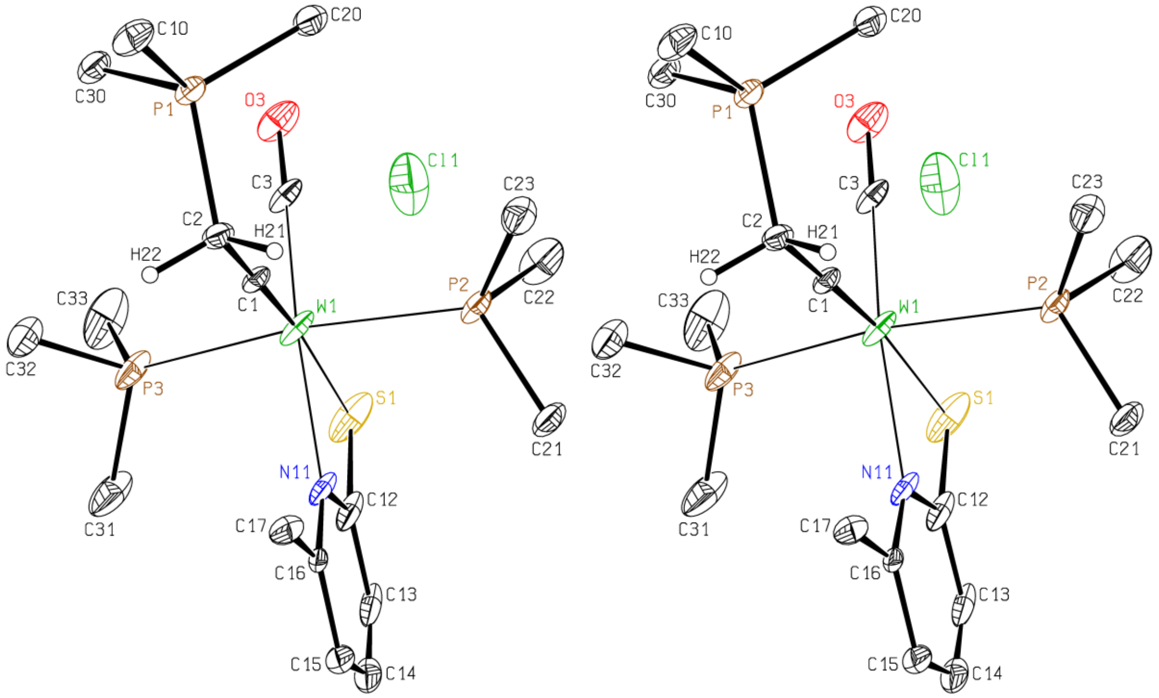

Figure S5. Stereoscopic ORTEP[8] plot of the ion pair containing the metal complex cation of **4** showing the atomic numbering scheme. The probability ellipsoids are drawn at the 20% probability level. The H atoms of the ethylidyne group are drawn with arbitrary radii, the other H atoms were omitted for clarity.

Table S8. Selected bond lengths (Å) and angles (°) for **4**.

W1–C1	1.793(4)	P3–C33	1.823(7)
W1–C3	1.992(5)	S1–C12	1.736(7)
W1–N11	2.221(4)		
W1–P2	2.4716(14)	C1–W1–S1	170.91(13)
W1–P3	2.4804(15)	C3–W1–N11	162.71(18)
W1–S1	2.6605(16)	P2–W1–P3	169.79(5)
C1–C2	1.486(6)	C2–C1–W1	179.4(4)
C2–P1	1.795(4)	C1–C2–P1	114.1(3)
P1–C10	1.772(5)	O3–C3–W1	179.1(4)
P1–C20	1.761(5)	C12–S1–W1	78.71(18)
P1–C30	1.779(5)	C16–N11–C12	120.7(4)
C3–O3	1.152(5)	C16–N11–W1	135.0(3)
P2–C21	1.827(4)	C12–N11–W1	104.3(4)
P2–C22	1.812(5)		
P2–C23	1.807(6)	C1–W1–N11–C16	–1.4(6)
P3–C31	1.822(6)	C3–W1–S1–C12	179.8(2)
P3–C32	1.822(6)	C1–C2–P1–C10	177.6(3)

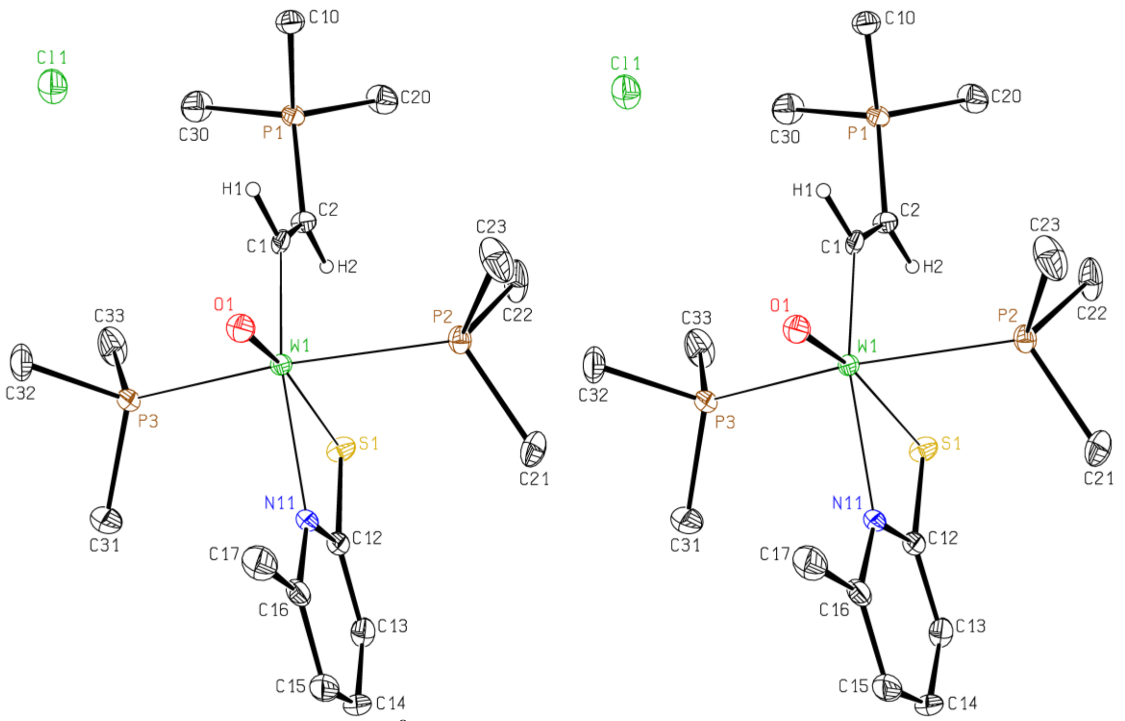

Figure S6. Stereoscopic ORTEP[8] plot of **5** showing the atomic numbering scheme. The probability ellipsoids are drawn at the 50% probability level. The H atoms of the ethenyl group are drawn with arbitrary radii, the other H atoms were omitted for clarity.

Table S9. Selected bond lengths (Å) and angles (°) for **5**.

W1–O1	1.715(2)	O1–W1–S1	167.89(7)
W1–C1	2.068(3)	C1–W1–N11	150.47(10)
W1–N11	2.190(3)	P2–W1–P3	160.56(3)
W1–P2	2.4974(8)	C2–C1–W1	135.8(2)
W1–P3	2.4948(9)	C2–C1–H1	114.4(6)
W1–S1	2.6931(8)	W1–C1–H1	109.7(6)
C1–C2	1.363(4)	C1–C2–P1	123.4(2)
C2–P1	1.745(3)	C1–C2–H2	122.0(6)
P1–C10	1.774(3)	P1–C2–H2	114.6(5)
P1–C20	1.794(3)	C12–S1–W1	78.67(11)
P1–C30	1.796(3)	C16–N11–C12	119.3(3)
P2–C22	1.811(3)	C16–N11–W1	133.4(2)
P2–C23	1.815(4)	C12–N11–W1	107.27(19)
P2–C21	1.815(3)		
P3–C33	1.815(3)	W1–C1–C2–P1	179.31(16)
P3–C32	1.816(3)	C1–C2–P1–C10	21.3(3)
P3–C31	1.818(4)	C2–C1–W1–S1	–1.7(3)
S1–C12	1.742(3)	C2–C1–W1–O1	177.6(3)
		C1–W1–S1–C12	179.3(2)
		O1–W1–N11–C12	–178.37(19)
		O1–W1–N11–C16	0.7(4)

4 NMR Spectra

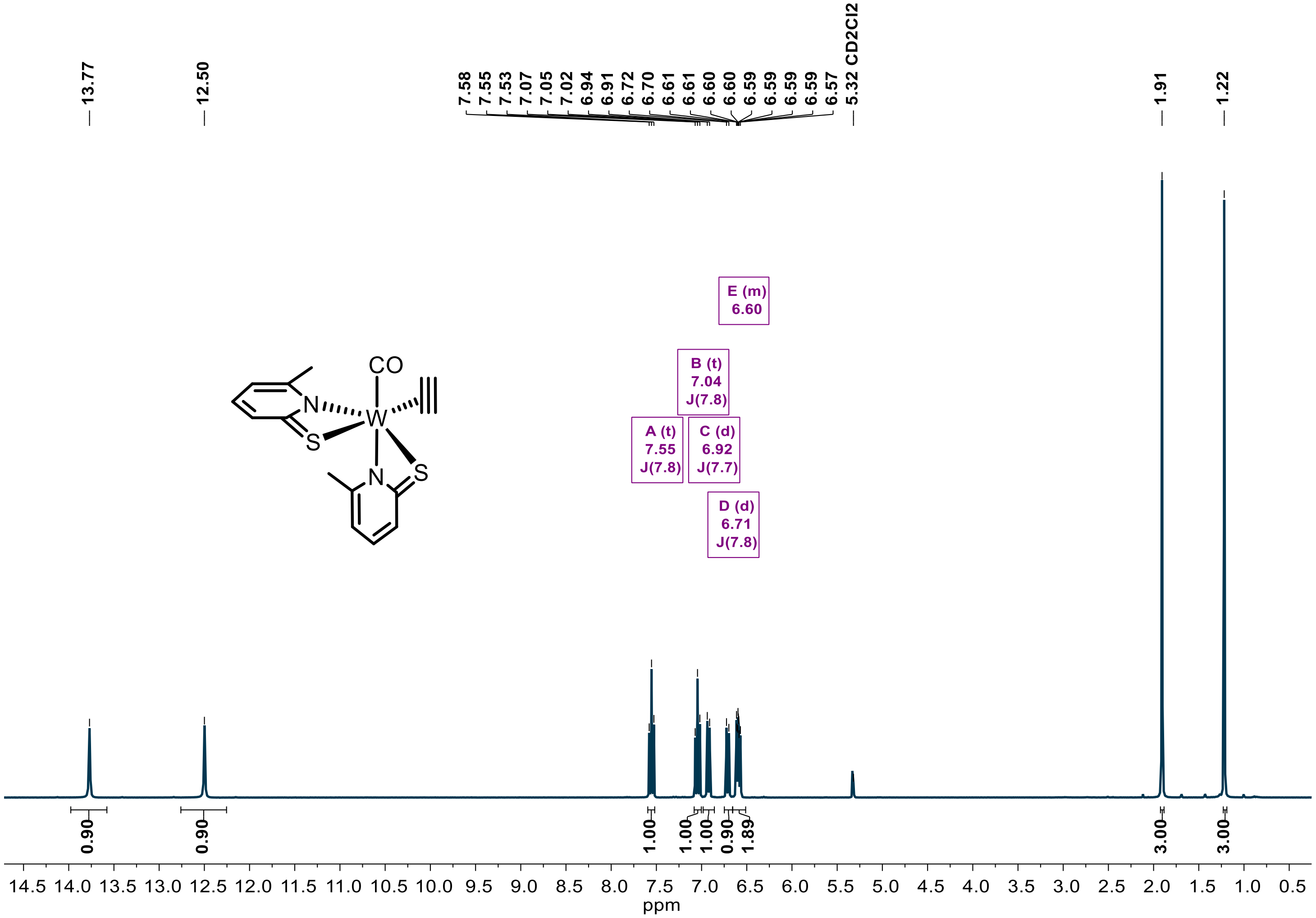

Figure S7. ^{1}H NMR spectrum of **1** in CD_2Cl_2.

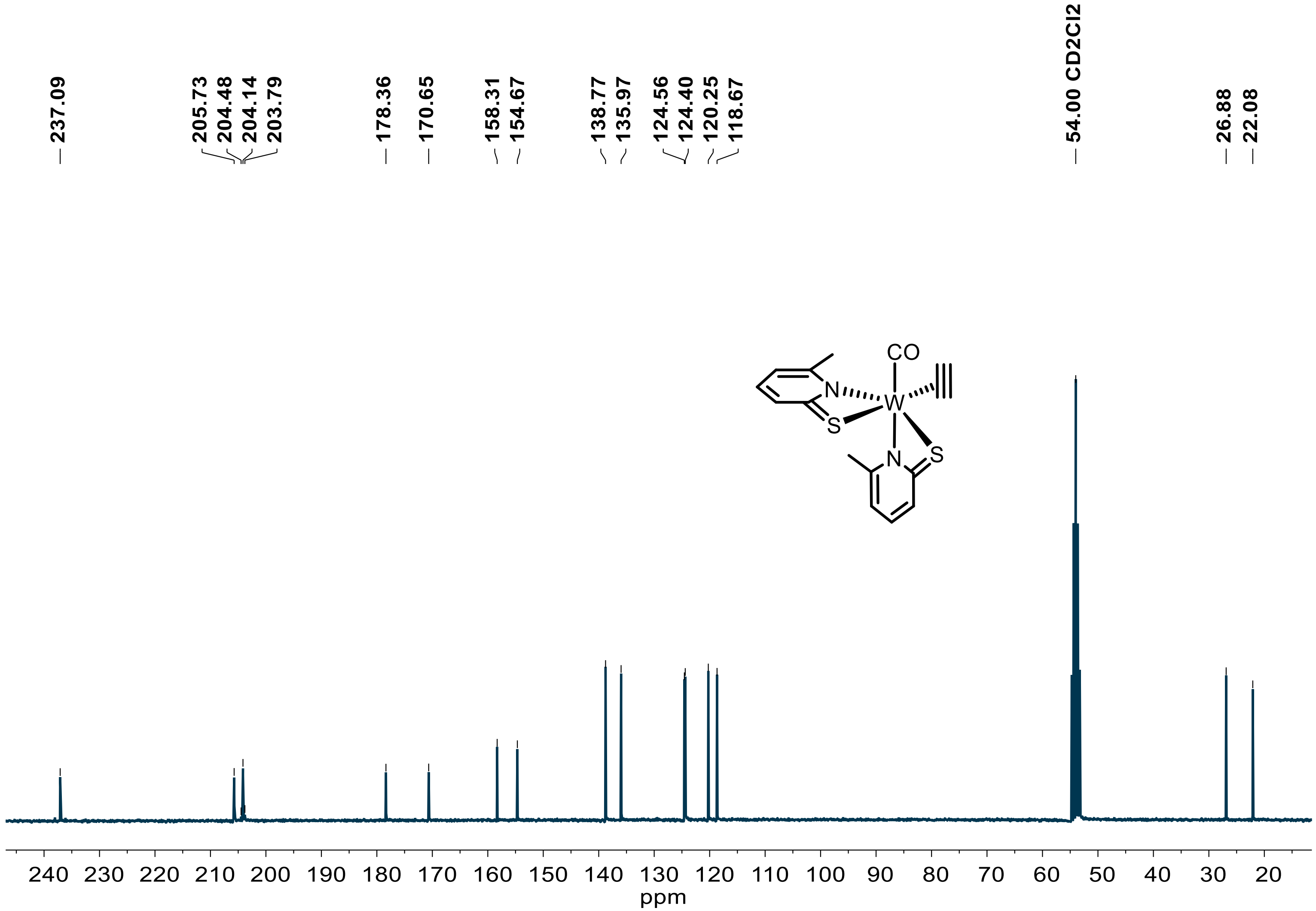

Figure S8. ^{13}C NMR spectrum of **1** in CD_2Cl_2.

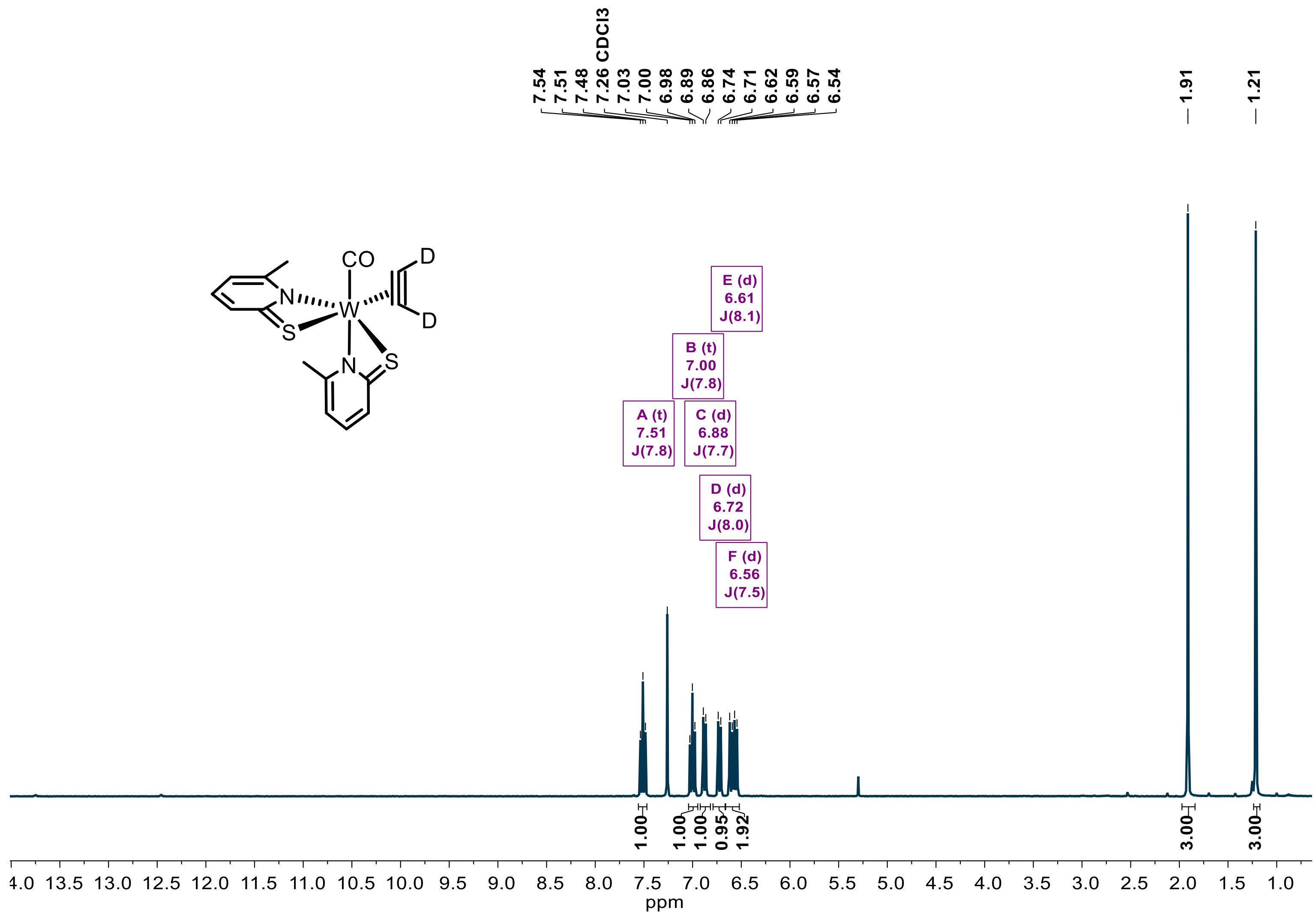

Figure S9. ^{1}H NMR spectrum of **1D** in $CDCl_3$.

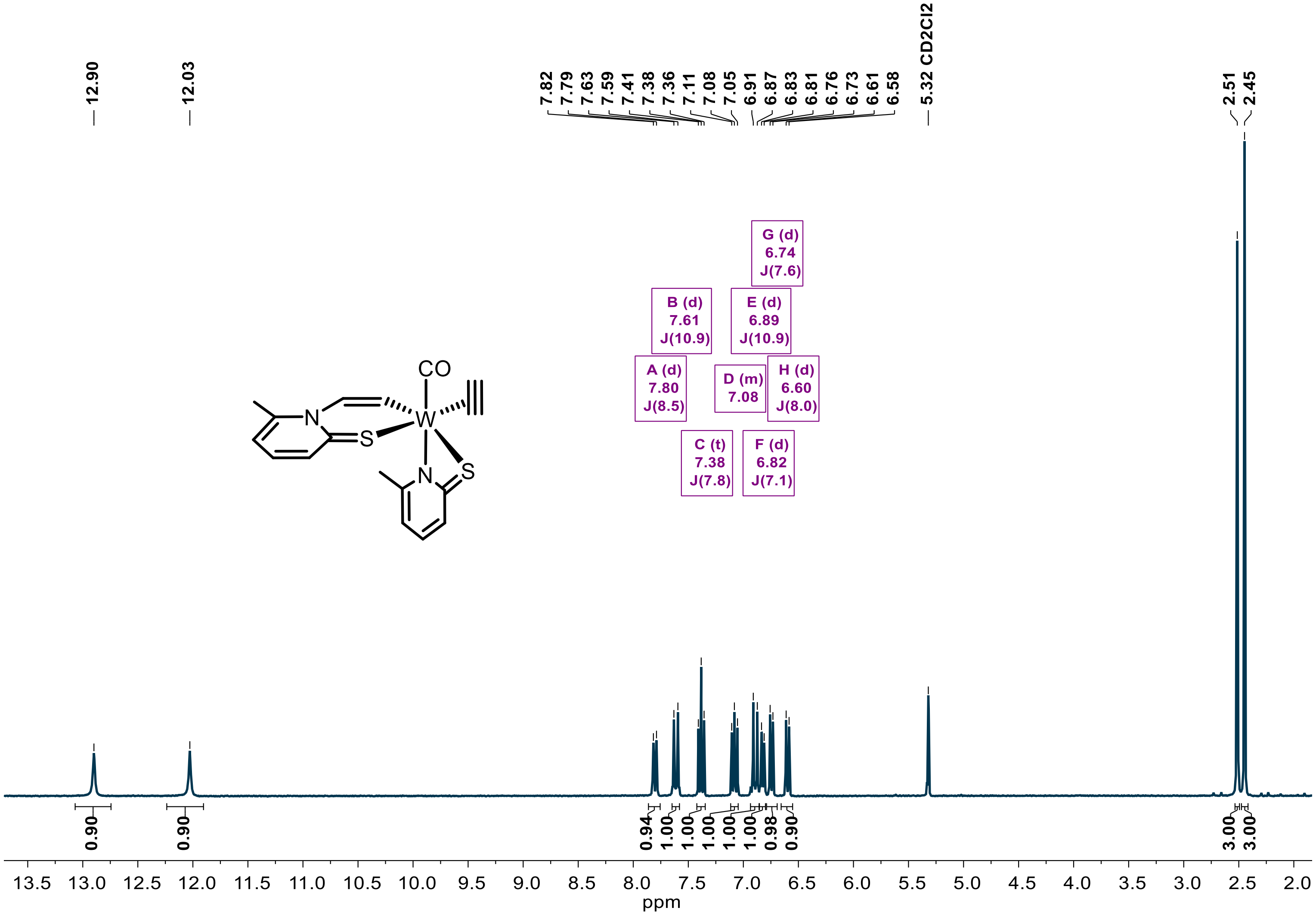

Figure S10. ^{1}H NMR spectrum of **2** in CD_2Cl_2.

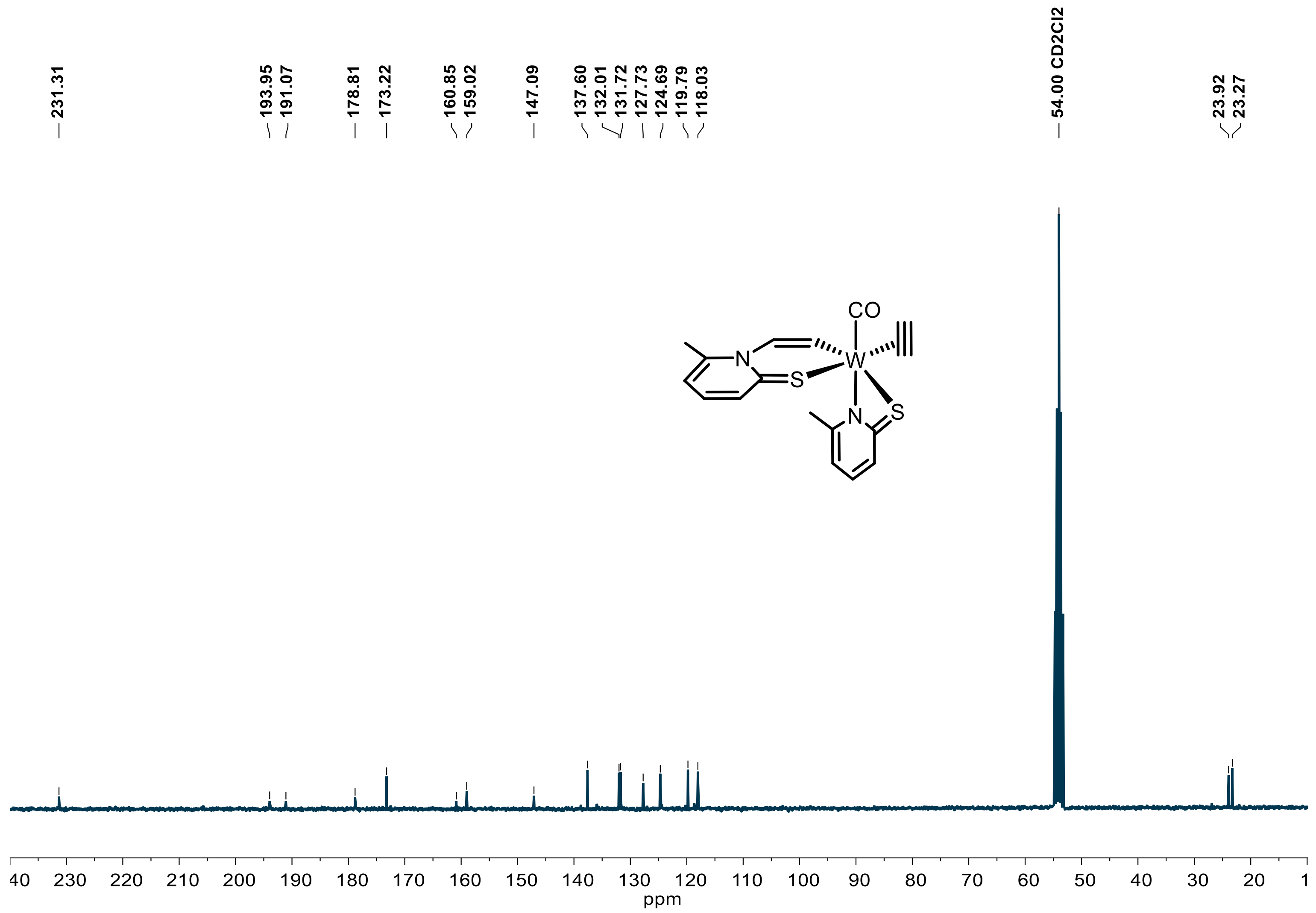

Figure S11. ^{13}C NMR spectrum of **2** in CD_2Cl_2.

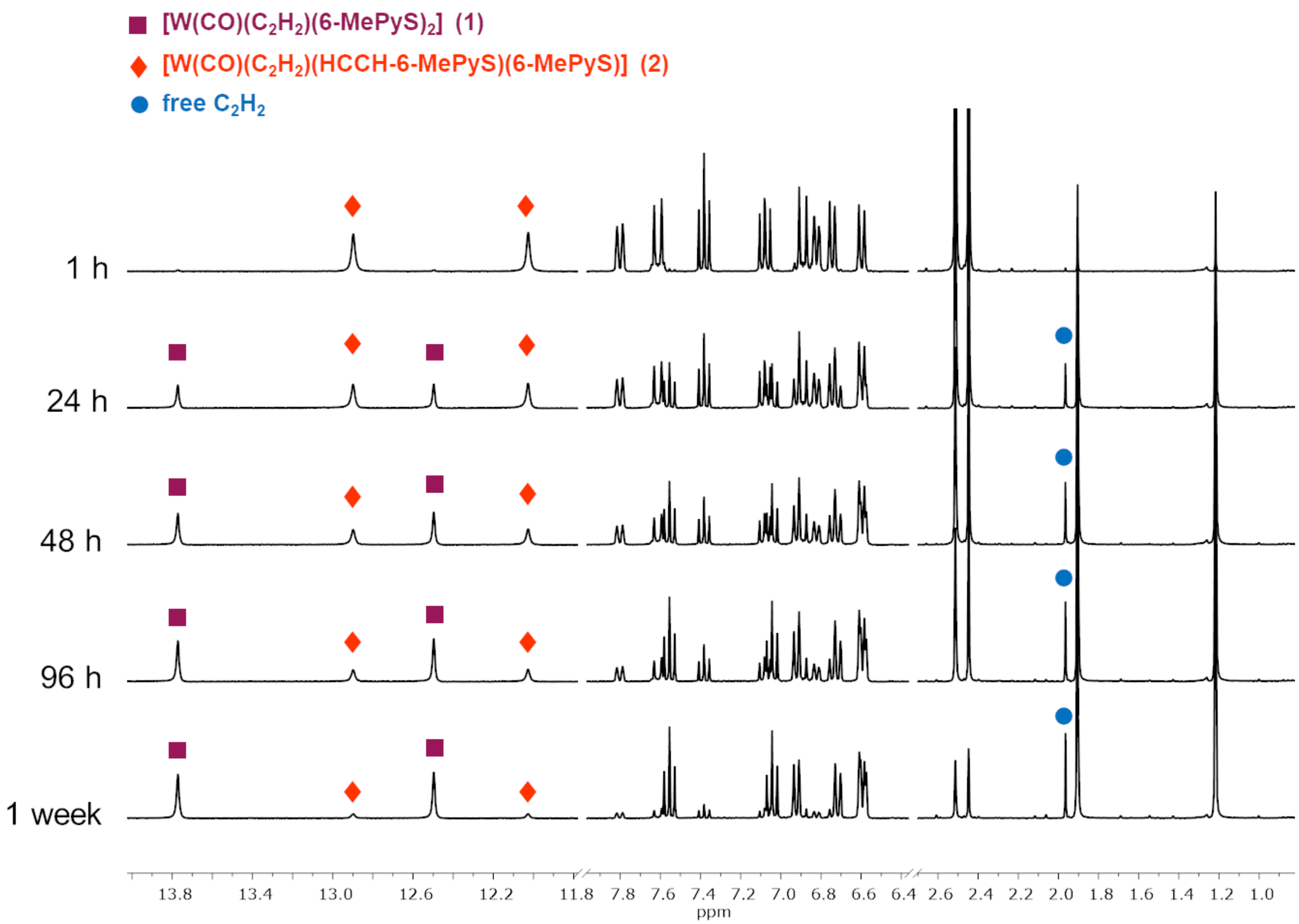

Figure S12. Decomposition of **2** to **1** under release of acetylene in CD_2Cl_2 followed by ^{1}H NMR spectroscopy for one week.

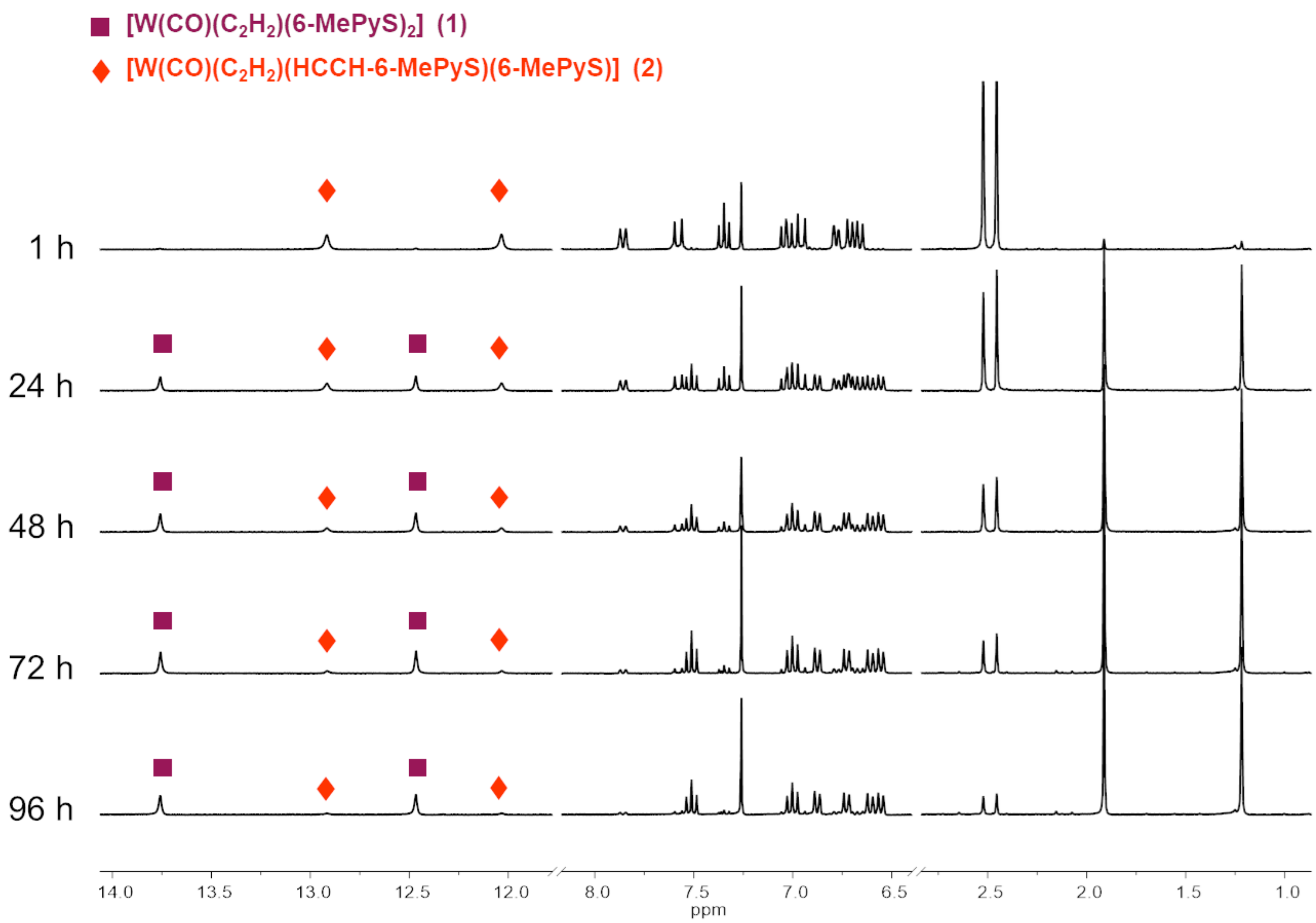

Figure S13. Decomposition of **2** to **1** under formation of polyacetylene in $CDCl_3$ followed by ^1H NMR spectroscopy for 96 h.

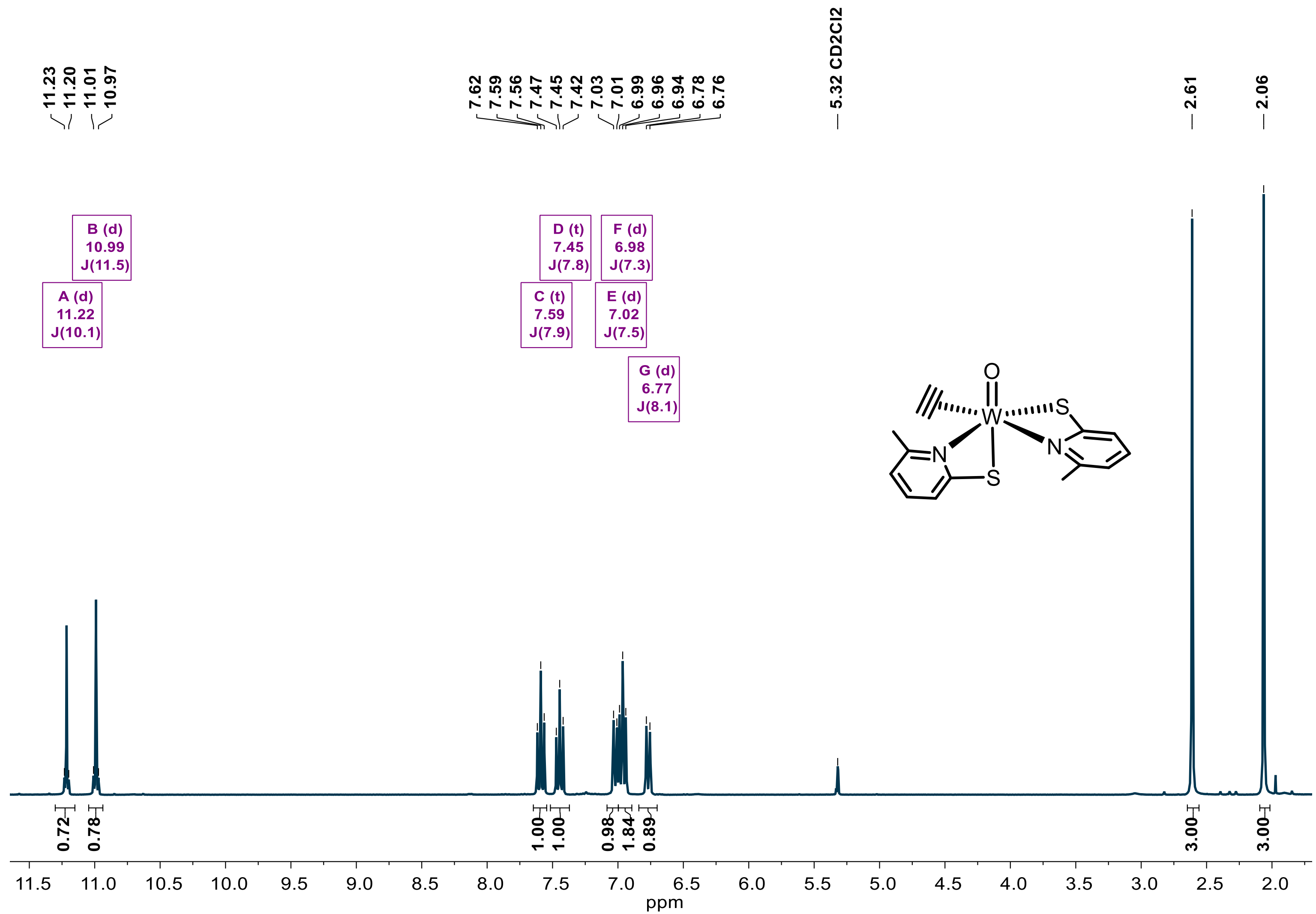

Figure S14. ^{1}H NMR spectrum of **3** in CD_2Cl_2.

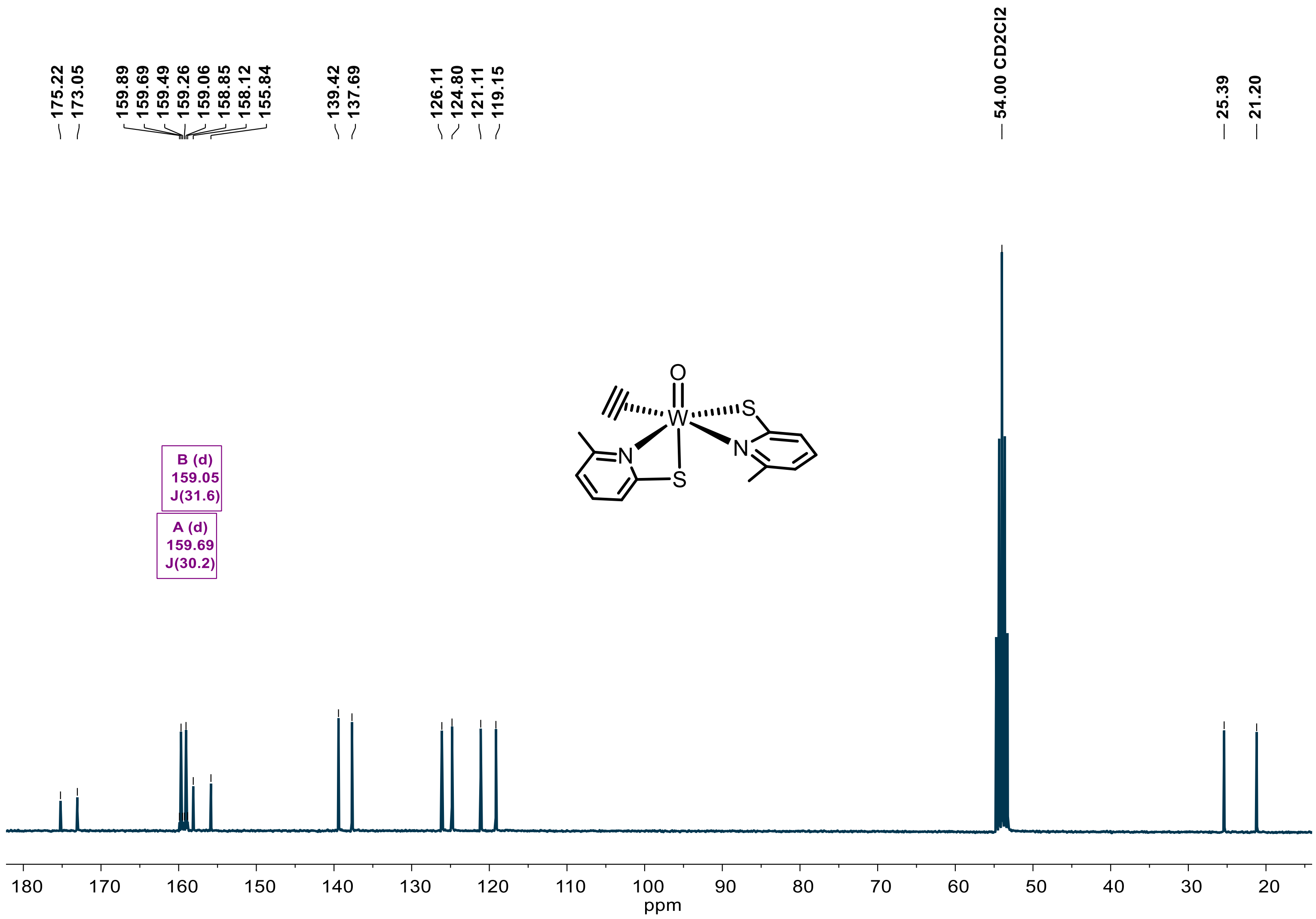

Figure S15. ^{13}C NMR spectrum of **3** in CD_2Cl_2.

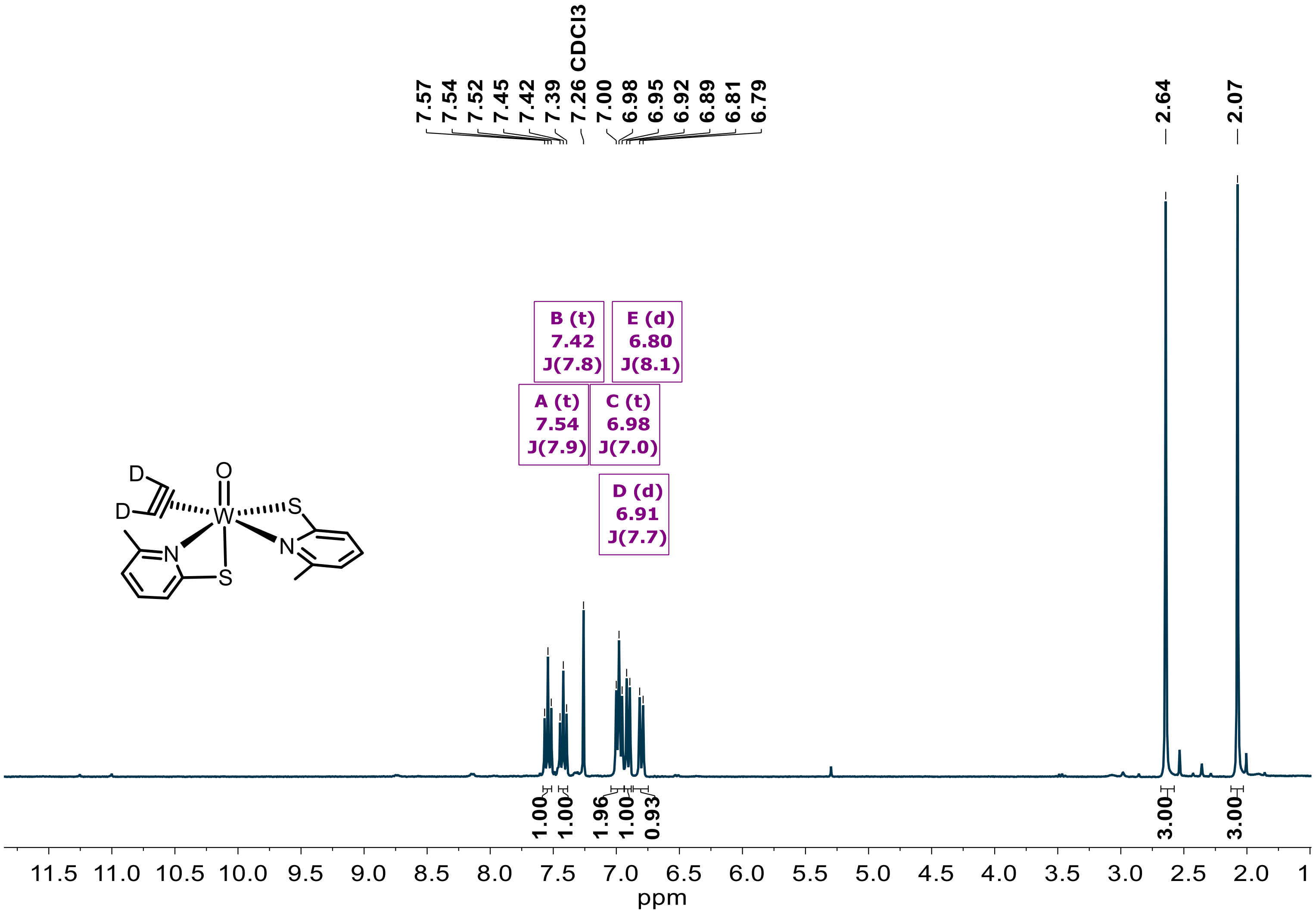

Figure S16. ^{1}H NMR spectrum of **3D** in $CDCl_3$.

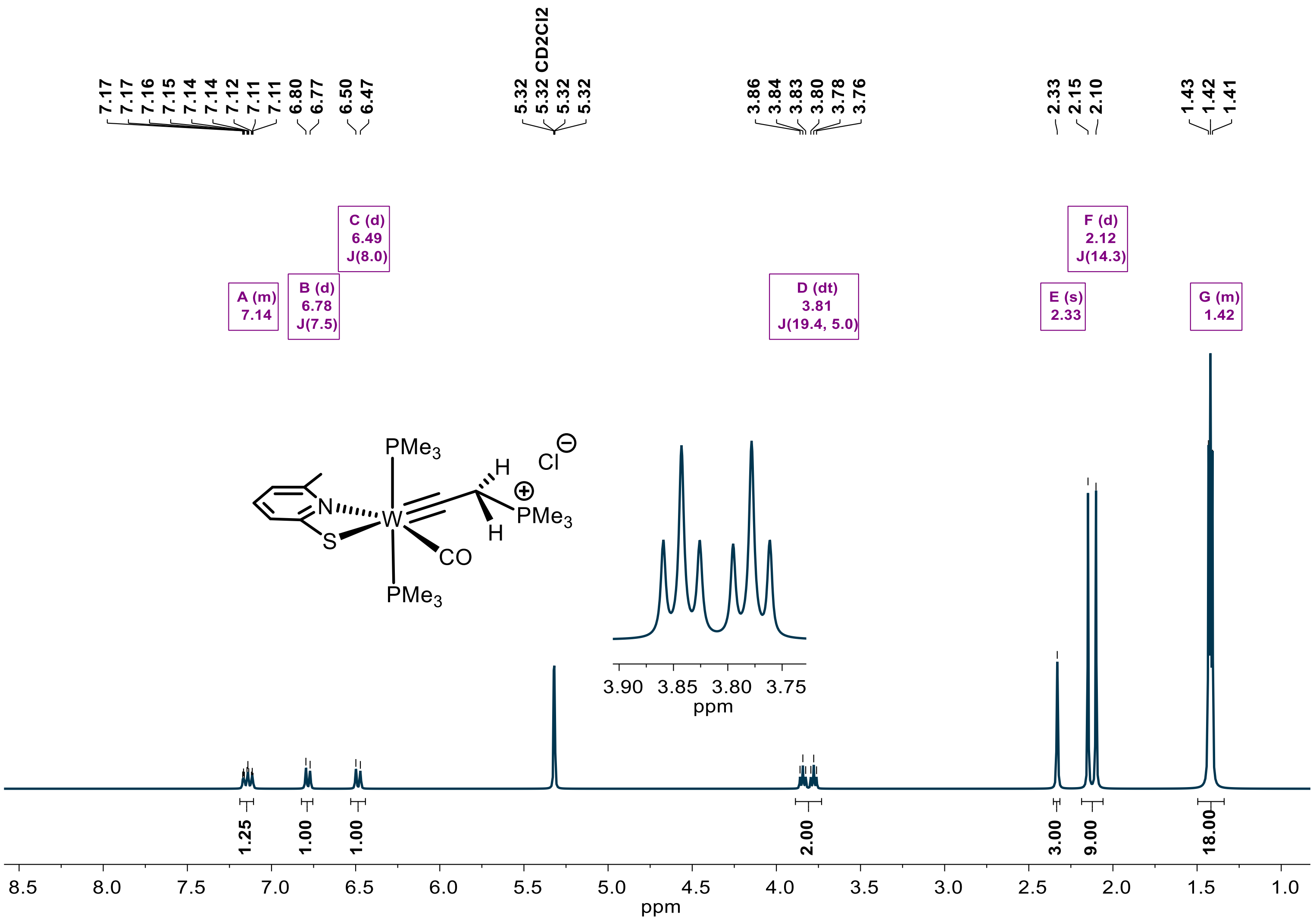

Figure S17. ^{1}H NMR spectrum of **4** in CD_2Cl_2.

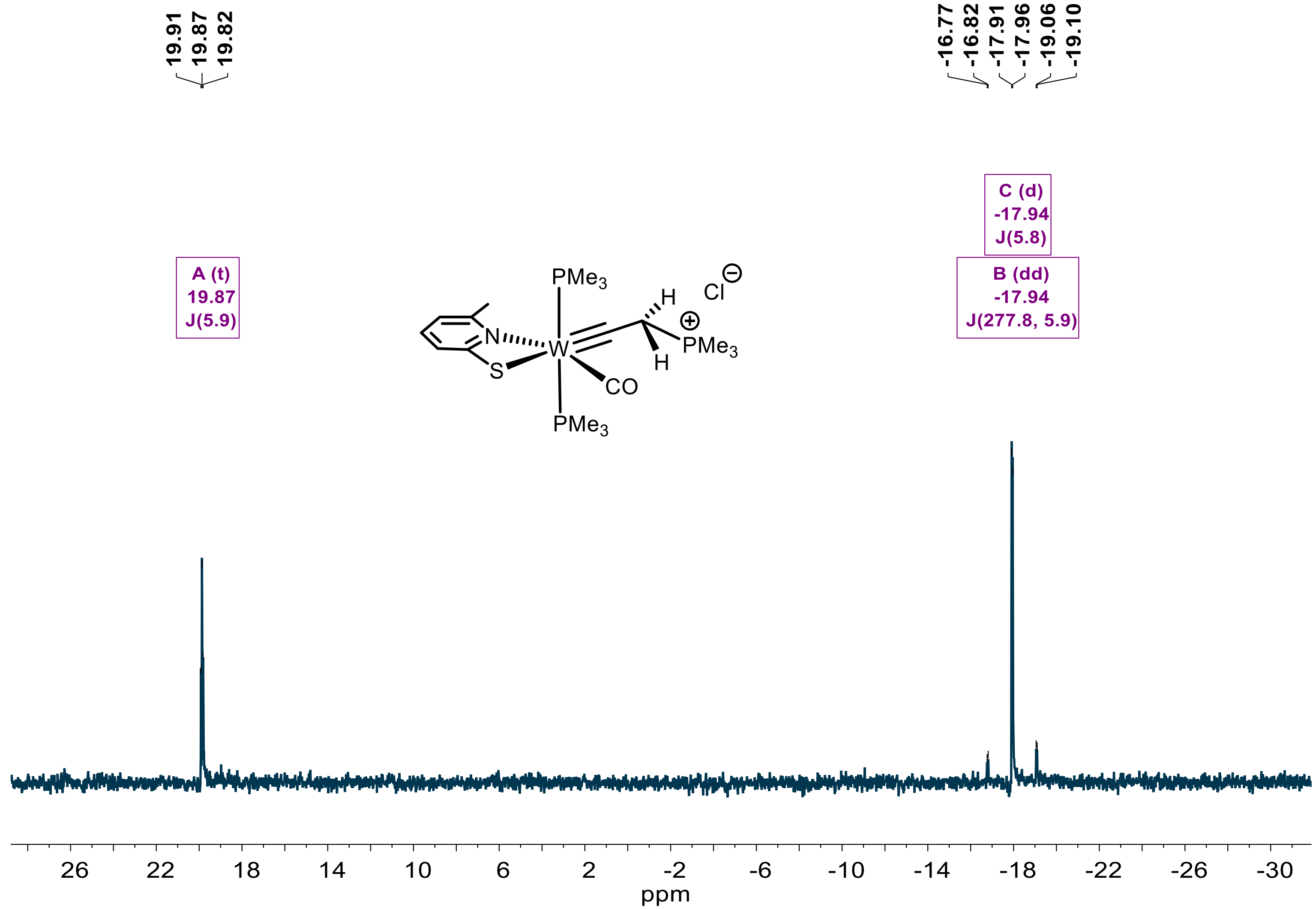

Figure S18. $^{31}P\{^{1}H\}$ NMR spectrum of **4** in CD_2Cl_2.

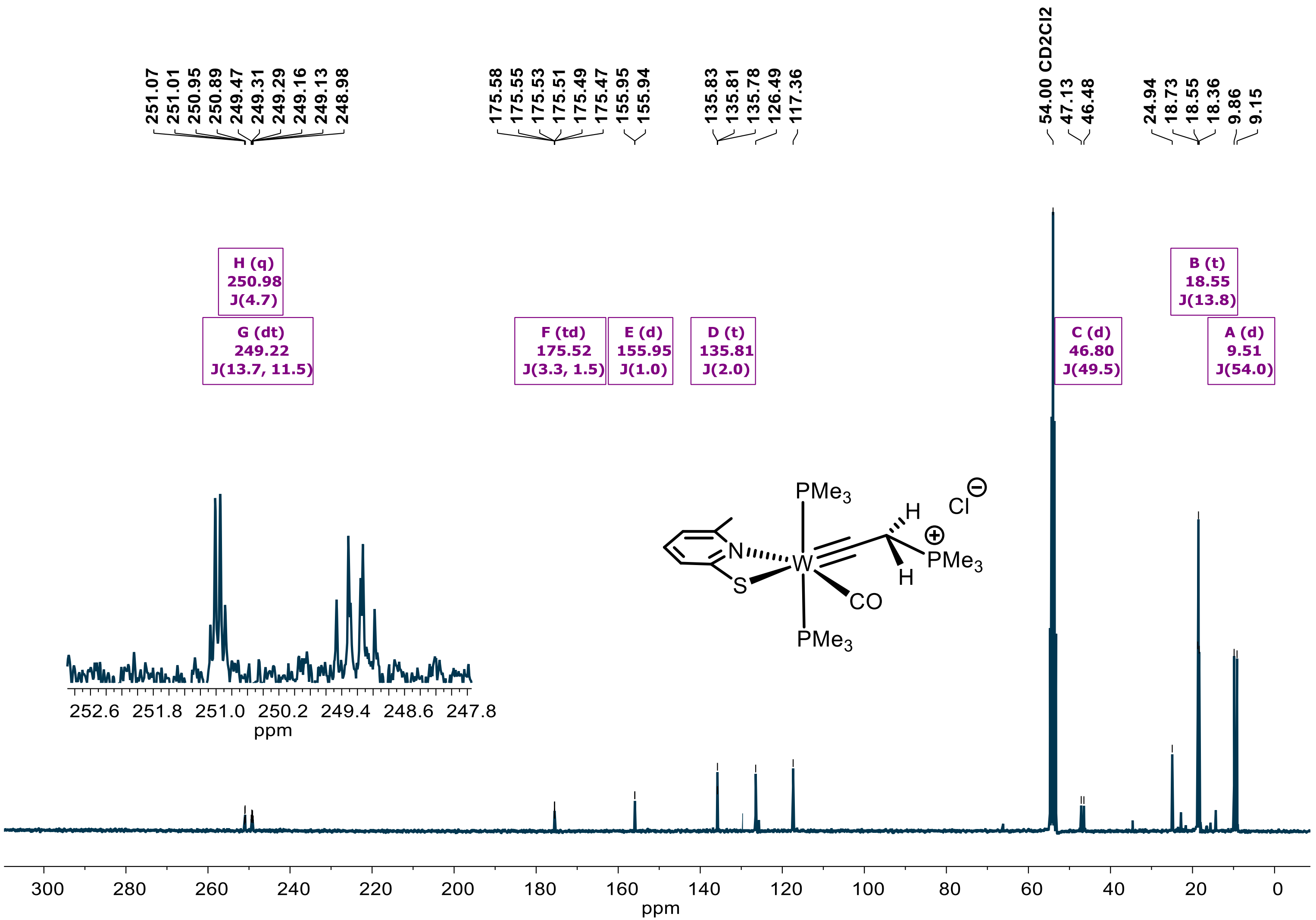

Figure S19. ^{13}C NMR spectrum of **4** in CD_2Cl_2. Extra signals come from toluene, Et_2O and pentane.

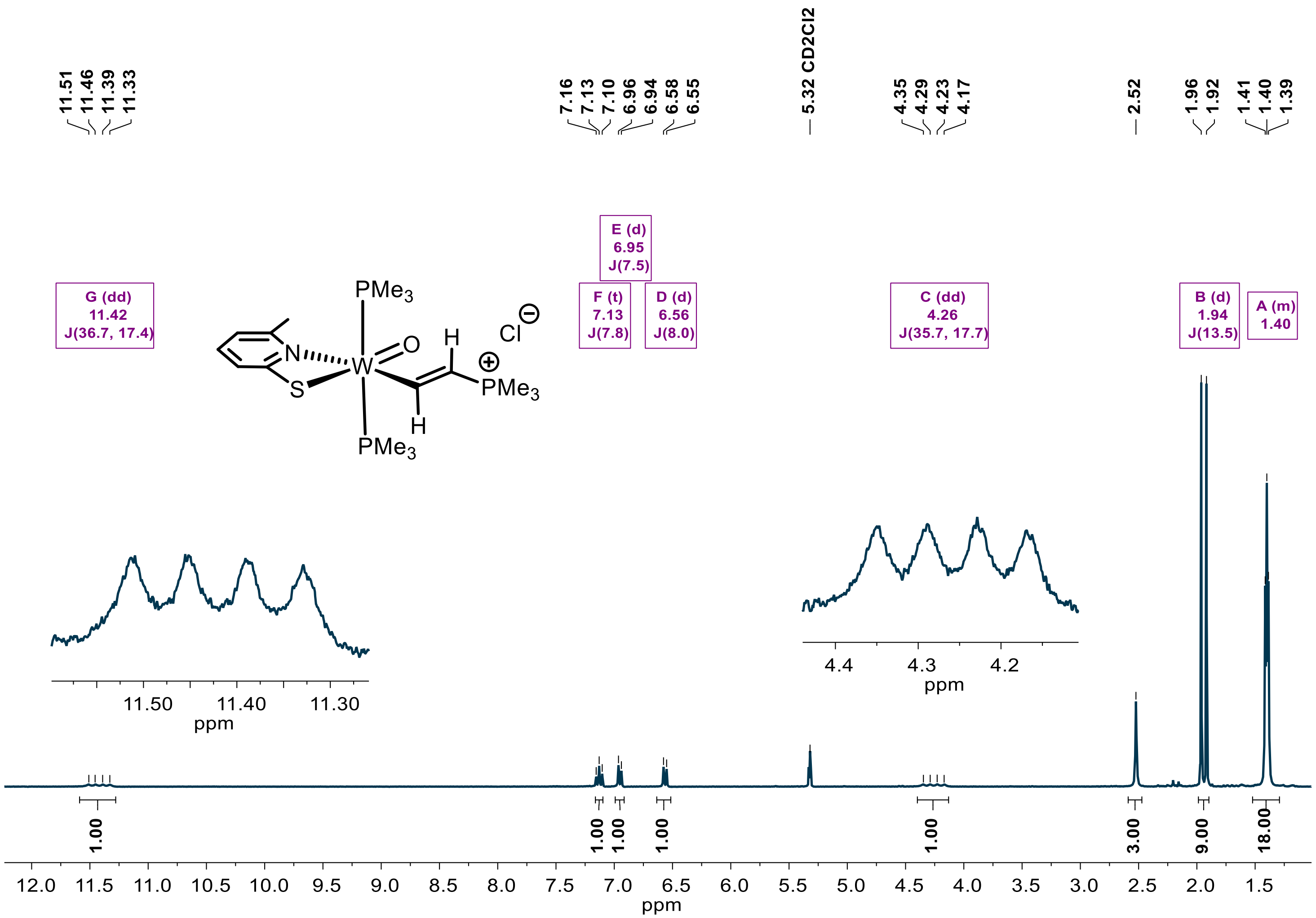

Figure S20. ^{1}H NMR spectrum of **5** in CD_2Cl_2.

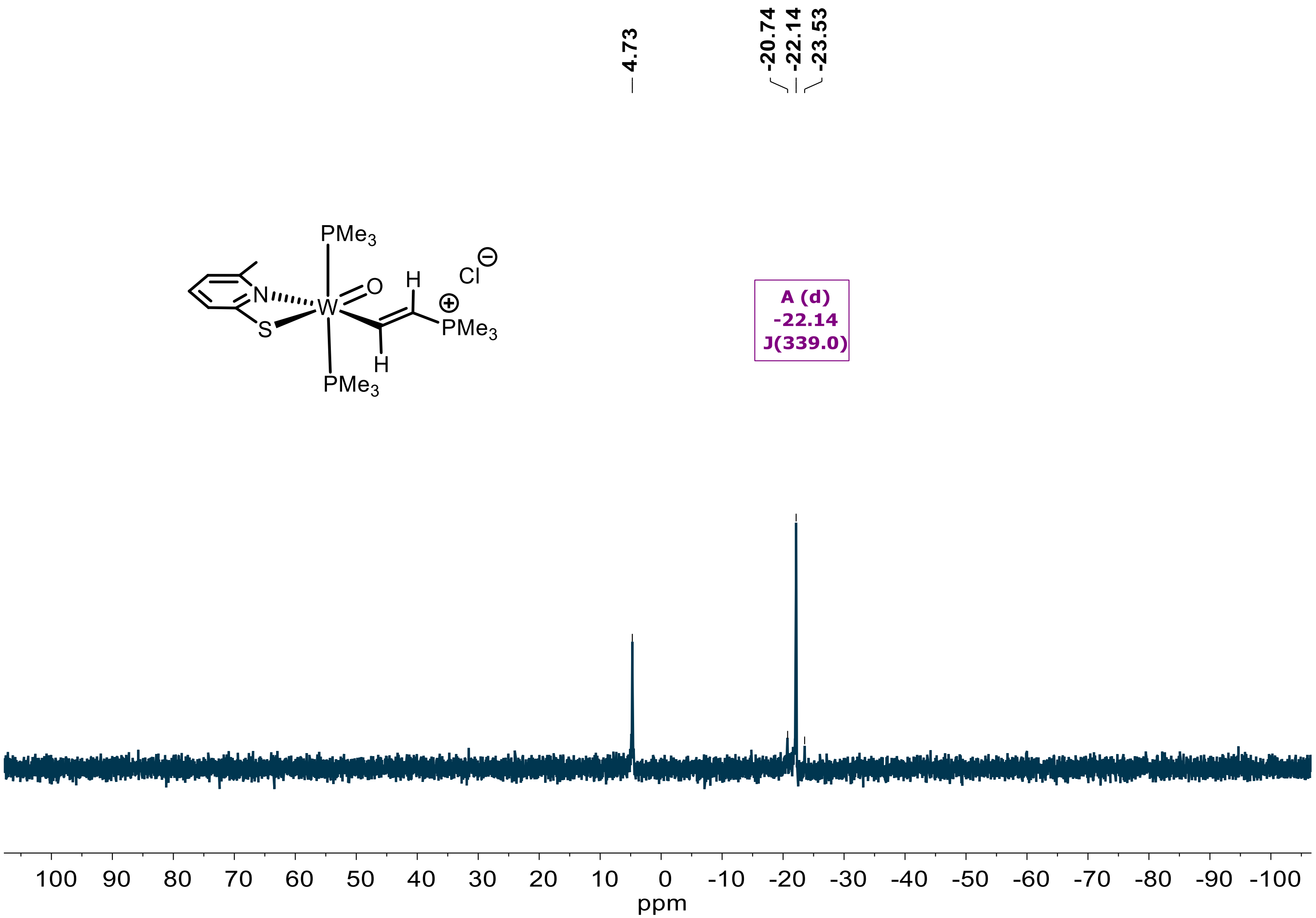

Figure S21. $^{31}P\{^{1}H\}$ NMR spectrum of **5** in CD_2Cl_2.

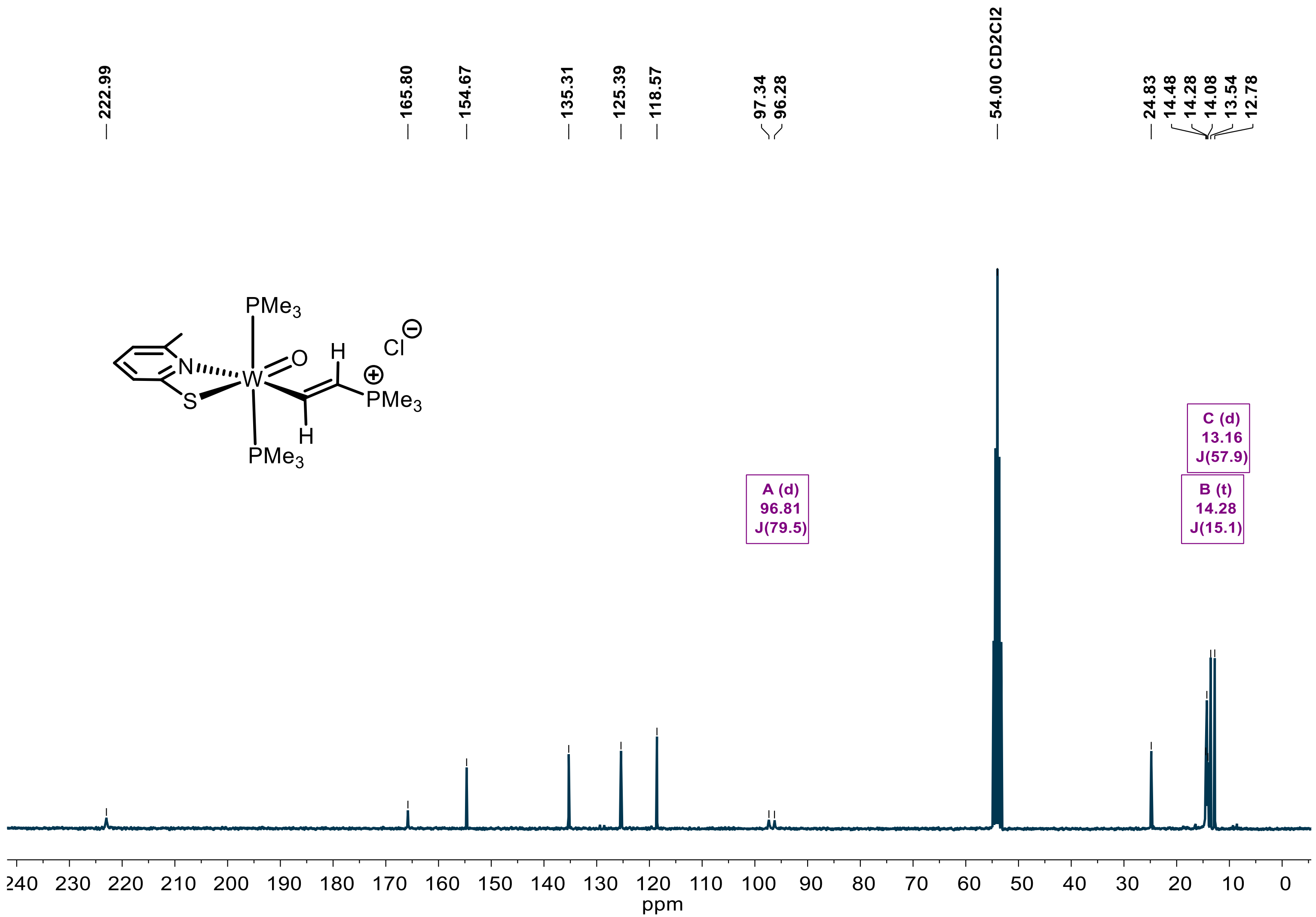

Figure S22. ^{13}C NMR spectrum of **5** in CD_2Cl_2.

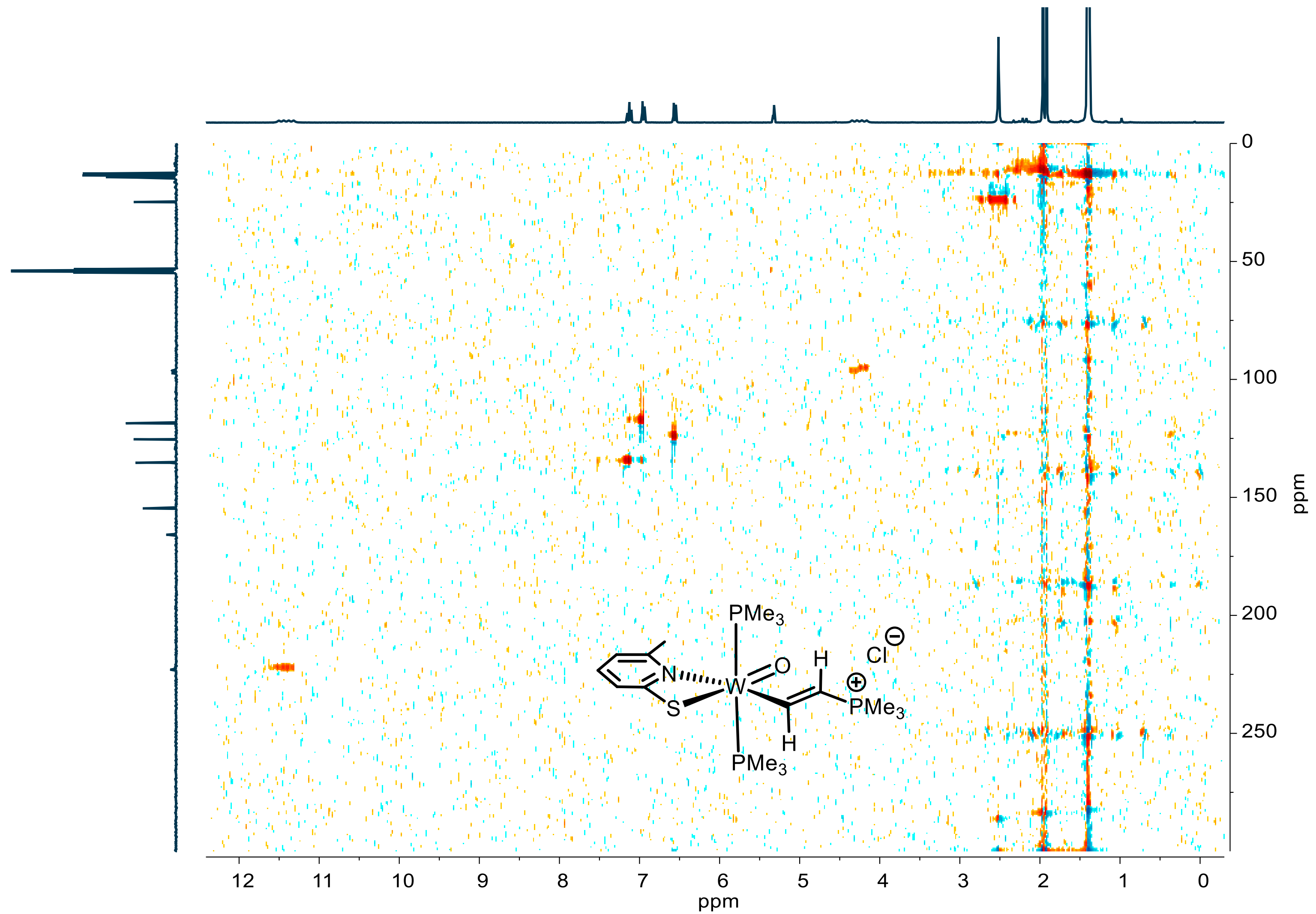

Figure S23. HSQC spectrum of **5** in CD_2Cl_2.

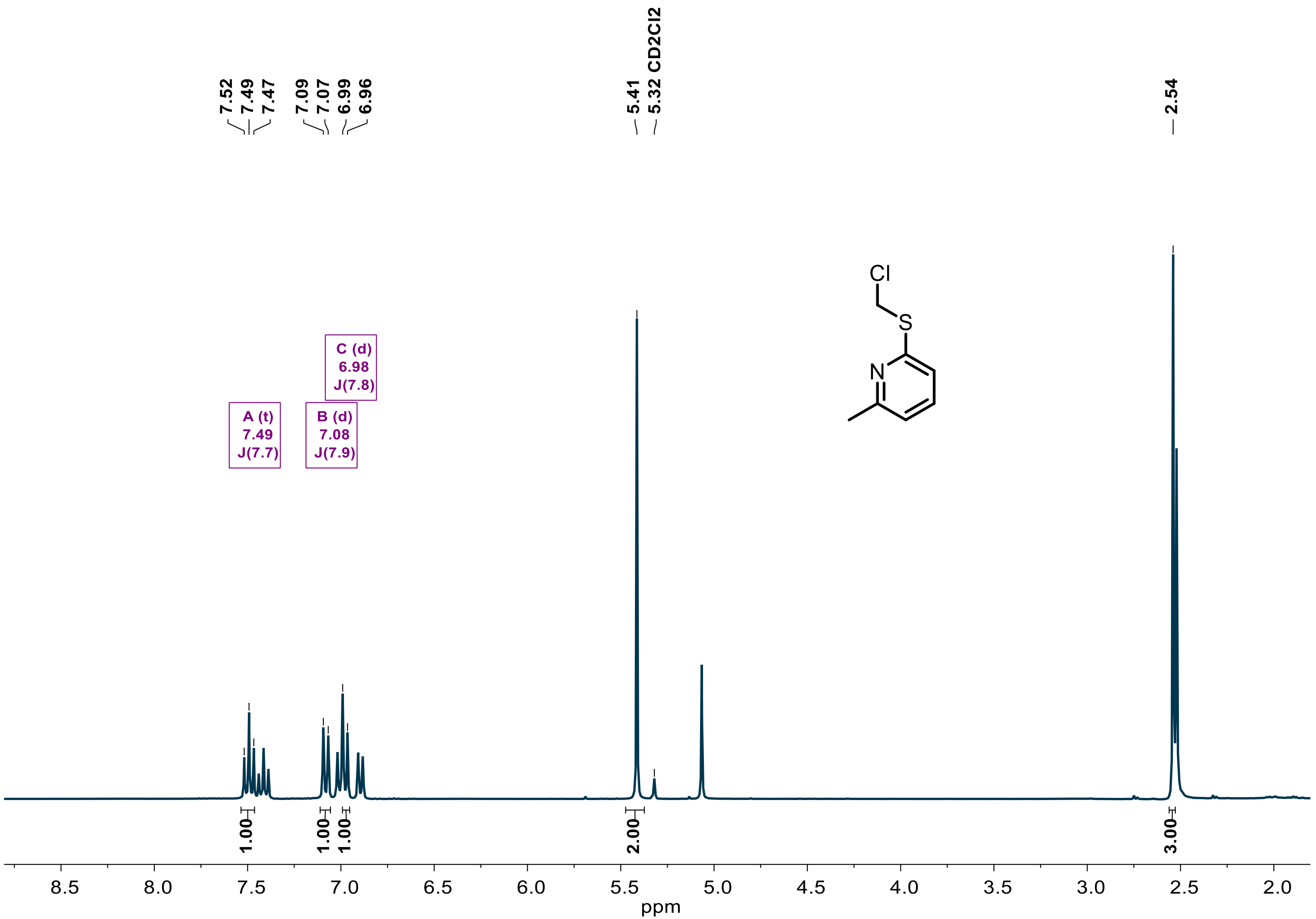

Figure S24. ^{1}H NMR spectrum of a mixture of 6-MePySCH$_2$Cl and (6-MePyS)$_2$CH$_2$ in CD$_2$Cl$_2$. Here, only peaks belonging to 6-MePySCH$_2$Cl are assigned.

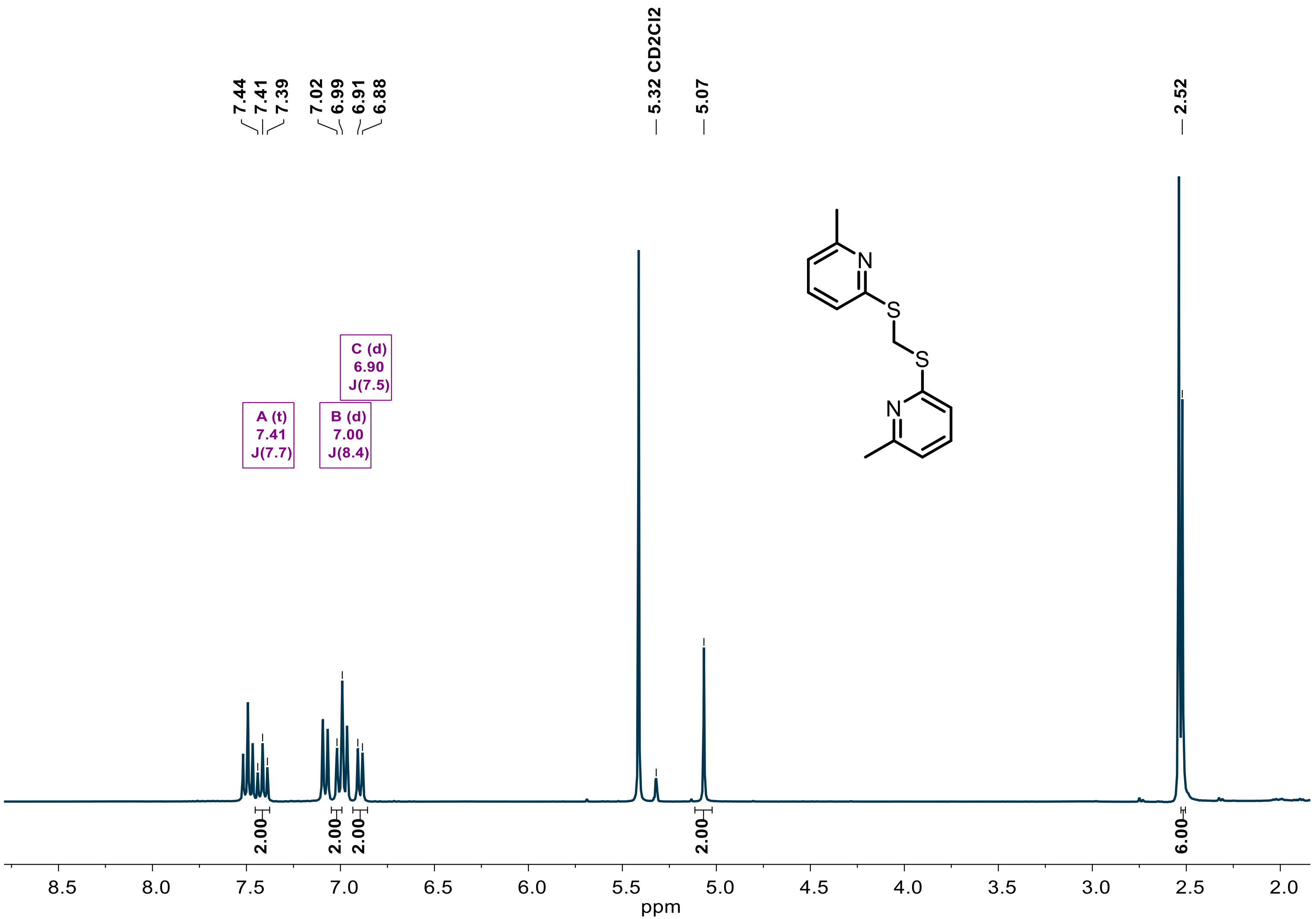

Figure S25. ^{1}H NMR spectrum of a mixture of 6-MePySCH$_2$Cl and (6-MePyS)$_2$CH$_2$ in CD$_2$Cl$_2$. Here, only peaks belonging to (6-MePyS)$_2$CH$_2$ are assigned.

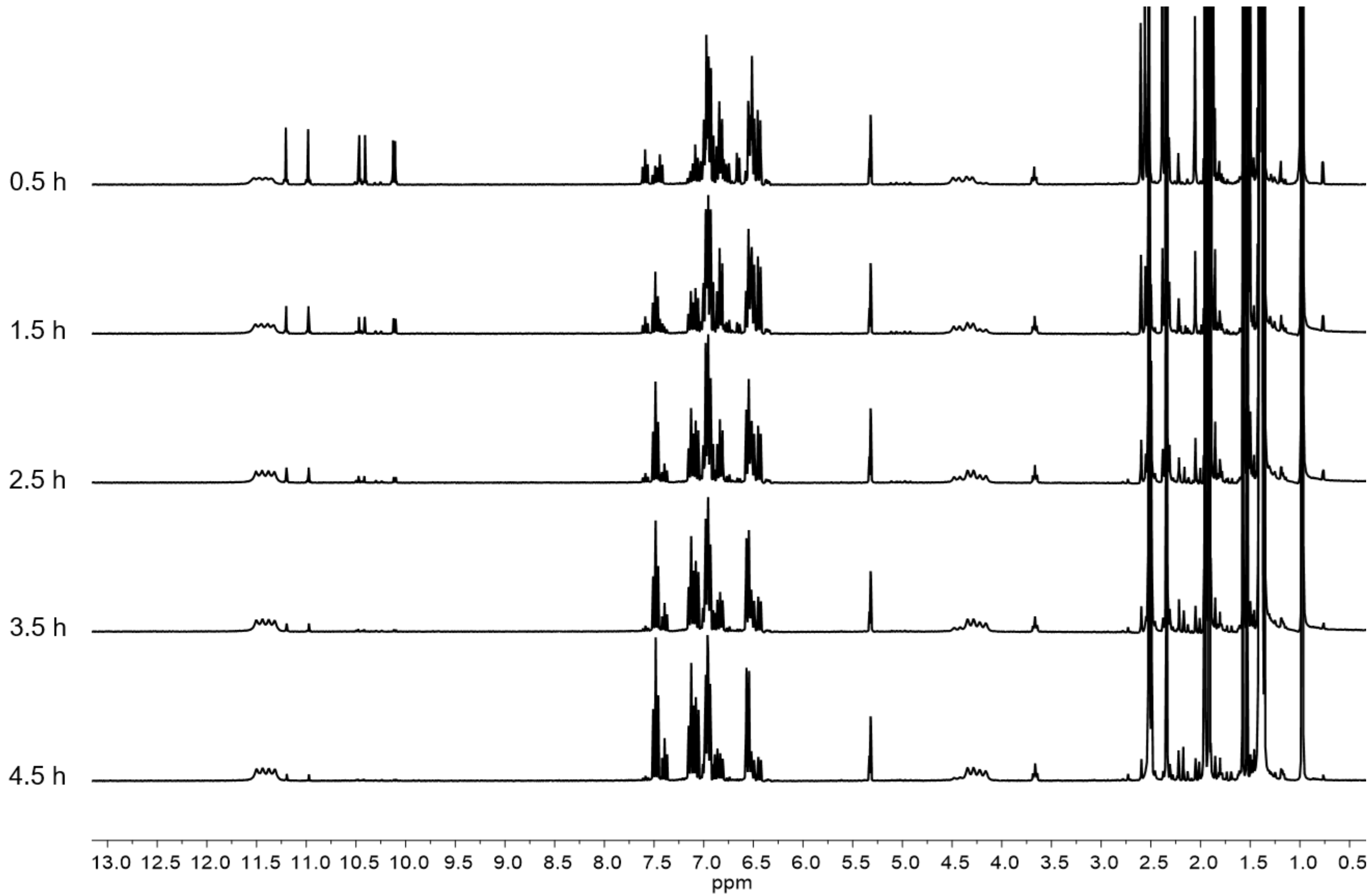

Figure S26. Reaction of **3** with 3.5 equiv of PMe_3 in 0.5 mL of CD_2Cl_2 in a J. Young NMR tube followed by ^1H NMR spectroscopy for 4.5 h.

5 References

(1) Vidovič, C.; Peschel, L. M.; Buchsteiner, M.; Belaj, F.; Mösch-Zanetti, N. C. Structural Mimics of Acetylene Hydratase: Tungsten Complexes Capable of Intramolecular Nucleophilic Attack on Acetylene. *Chem. Eur. J.* **2019**, *25* (63), 14267–14272.

(2) Kanishchev, O. S.; Dolbier, W. R. Synthesis and Characterization of 2-Pyridylsulfur Pentafluorides. *Angew. Chem. Int. Ed.* **2015**, *54*, 280–284.

(3) Adger, B. M.; Ayrey, P.; Bannister, R.; Forth, M. A.; Hajikarimian, Y.; Lewis, N. J.; O'Farrell, C.; Owens, N.; Shamji, A. Synthesis of 2-substituted 4-pyridylpropionates. Part 2. Alkylation approach. *J. Chem. Soc., Perkin Trans. 1* **1988**, *10*, 2791–2796.

(4) Ehweiner, M. A.; Wiedemaier, F.; Belaj, F.; Mösch-Zanetti, N. C. Oxygen Atom Transfer Reactivity of Molybdenum(VI) Complexes Employing Pyrimidine- and Pyridine-2-thiolate Ligands. *Inorg. Chem.* **2020**, *59*, 14577–14593.

(5) Sheldrick, G. M. A short history of SHELX. *Acta Cryst. A* **2008**, *64* (Pt 1), 112–122.

(6) Sheldrick, G. M. Crystal structure refinement with SHELXL. *Acta Cryst. C* **2015**, *71* (Pt 1), 3–8.

(7) Spek, A. L. PLATON SQUEEZE: A tool for the calculation of the disordered solvent contribution to the calculated structure factors. *Acta Cryst. C* **2015** (71), 9–18.

(8) Johnson, C. K. *ORTEP. Report ORNL-3794.*, 1965.
